# Multi-omics analyses reveal the defense mechanisms behind the tolerance of the ‘Parson Brown’ sweet orange to Huanglongbing

**DOI:** 10.1186/s12870-025-07372-2

**Published:** 2025-10-03

**Authors:** Lamiaa M. Mahmoud, Shelley E. Jones, Pedro Gonzalez-Blanco, Yu Fahong, Manjul Dutt, Nabil Killiny

**Affiliations:** 1 Department of Plant Pathology, Citrus Research and Education Center, IFAS, University of Florida, 700 Experiment Station Road, Lake Alfred, FL 33850 USA; 2 Interdisciplinary Center for Biotechnology Research (UF | ICBR), University of Florida, Gainesville, FL USA; 3 Department of Horticultural Sciences, Citrus Research and Education Center, University of Florida, Lake Alfred, FL USA; 4 Plant Breeding Graduate Program, University of Florida, Gainesville, FL USA

**Keywords:** Citrus greening, Huanglongbing, Sweet orange, Terpene biosynthesis, Metabolomics, Transcriptomics, Disease tolerance

## Abstract

**Background:**

‘Parson Brown’ sweet orange is an early-maturing variety and is considered a resilient tree in the face of Huanglongbing (Citrus Greening) disease. Its ability to maintain productivity under endemic HLB conditions has demonstrated its value for growers battling this devastating disease. This study compared the metabolomic profile, transcriptomic analysis, and physiological responses of three early-maturing sweet oranges: ‘Hamlin’, ‘Roble’, and ‘Parson Brown’.

**Results:**

Healthy greenhouse-grown trees were propagated and exposed to ‘*Candidatus* Liberibacter asiaticus’ via psyllid infestation. We recorded a decrease of landed psyllids on ‘Parson Brown’ (20.58%) compared to ‘Hamlin’ (34.38%) and ‘Roble’ (45.04%), in addition to a lower ‘*Ca*. L. asiaticus’ titer in ‘Parson Brown’. Transcriptomic profiling indicated cultivar-specific expression patterns, with ‘Parson Brown’ showing strong upregulation of genes involved in terpenoid and flavonoid biosynthesis. Infected ‘Parson Brown’ trees exhibited significantly higher total phenolic and flavonoid contents, lower ROS and H₂O₂ levels, and enhanced expression of antioxidant-related genes. Volatile analysis revealed distinct profiles in ‘Parson Brown’, including elevated levels of certain monoterpenes, which may contribute to reduced vector attraction.

**Conclusion:**

The tolerance of ‘Parson Brown’ is driven by a multifaceted defense response, emphasizing the value of traditional breeding in combining diverse resistance traits from parental lines.

**Supplementary Information:**

The online version contains supplementary material available at 10.1186/s12870-025-07372-2.

## Background

Citrus is a major global fruit crop, cultivated in over one hundred countries and playing a significant role in the agricultural economies of tropical and subtropical regions [1]. However, diseases and pests increasingly threaten citrus production, severely impacting fruit yield and tree health. In Florida, citrus greening disease, also known as Huanglongbing (HLB), is caused by the phloem-restricted bacterium ‘*Candidatus* Liberibacter asiaticus’, and transmitted by Asian citrus psyllid (ACP), *Diaphorina citri* Kuwayama. This disease represents the most significant challenge to citrus production worldwide [2].

HLB in Florida has been devastating, as the state was one of the largest citrus-producing regions in the United States and the world. The disease symptoms are characterized by leaf chlorosis, stunted growth, premature fruit drop, and malformed, bitter-tasting fruits that are unmarketable [3, 4]. Since HLB was detected in Florida in 2005, the state’s citrus industry has experienced dramatic production declines, with losses exceeding 92% of pre-HLB levels [5].

The sharp decline in citrus production has forced growers to adopt costly and labor-intensive strategies to manage HLB. Given the complex nature of the disease, a multi-faceted approach is essential. Current efforts include Vector-targeted chemical control, enhanced nutritional management, thermotherapy, and the application of antimicrobials and antibiotics to combat ‘*Ca*. L. asiaticus’ infection. Despite these efforts, tree health continues to deteriorate, and fruit yields continue declining, emphasizing the urgent need for effective and sustainable solutions.

Most sweet orange [*Citrus sinensis* [L.] Osbeck : [6] cultivars, which form the cornerstone of Florida’s citrus juice industry, are highly susceptible to HLB [7–11]. Despite this susceptibility, Florida citrus growers have observed some trees that exhibit remarkable resilience and sustained productivity even with prolonged exposure to HLB. The ‘Parson Brown’ sweet orange, has emerged as a promising candidate for tolerance compared to other susceptible varieties under similar disease pressure [10]. ‘Parson Brown’ originated as a chance seedling in the dooryard of Rev. N. L. Brown near Webster, Florida, and is said to have been planted in 1856 [12]. Historically, around fifteen clones of ‘Parson Brown’ were registered with the Florida Department of Agriculture Citrus Budwood Program in the mid-20th century. However, only one original clone, F-56-2, is still maintained by the Division of Plant Industry (DPI) [13, 14]. Another early sweet orange variety, Roble’, has demonstrated relative tolerance to HLB under field conditions. The clone, 502-4-12, originated from a clonal selection made at the Pless Nursery in Thonotosassa, Hillsborough County, is commercially grown, and entered the budwood program in 1970.

Metabolomics and transcriptomics approaches are powerful tools for identifying alterations in biological functions and metabolism in response to stresses [15]. Cellular metabolites are the final products of cellular regulation that are linked to the phenotype [16]. In recent years, metabolomics-based studies of the citrus HLB pathosystem have increased with a focus on mapping and identifying metabolites associated with HLB symptoms [17–20]. Modifications in the transcriptome directly influence the production of metabolites, linking gene activity to physiological functions and facilitating a deeper understanding of cellular responses to stress [9, 21].

Hence, we hypothesize that integrating metabolomic profiling, transcriptomic analysis, and physiological measurements will lead to identifying key defense mechanisms associated with the tolerance to *‘Ca.* L. asiaticus*’* in the HLB-tolerant sweet orange trees. We examined critical defense responses, including reactive oxygen species (ROS) regulation, callose deposition, starch accumulation, secondary metabolites alteration, and volatile emission of three early-season sweet orange varieties - ‘Hamlin’, ‘Roble’, and the resilient ‘Parson Brown’ upon infection with *‘Ca.* L. asiaticus*’*.

## Methods

### Plant materials and insect colonies

Budwoods of three sweet orange varieties (‘Hamlin’ [clone 1-4-1], ‘Roble’ [clone 502-4-12], and ‘Parson Brown’ [clone F-56-2]) were obtained from the Florida Department of Agriculture and Consumer Services (DPI) and stick-grafted onto Swingle citrumelo rootstock trees. Trees were fertilized using Harrell’s 16–5–10 nursery controlled-release fertilizer (CRF; Harrell’s LLC, USA). Trees were maintained in a certified greenhouse (27 ± 2 °C, 60 ± 5% relative humidity, and 16: 8 h L/D photocycle) at the Citrus Research and Education Center (CREC), University of Florida (28°10’N, 81°71’E), Lake Alfred, Florida. Following successful grafting, one-year-old trees were used in this study.

To obtain the ‘*Ca*. L. asiaticus’-infected colonies, *D. citri* from the healthy colonies, were reared on HLB-symptomatic and qPCR-positive ‘*Ca*. L. asiaticus’-infected alemow trees (*Citrus macrophylla*) inside 400-mesh cages (BioQuip, Rancho Dominguez, CA) and maintained in a secured growth room (27 ± 2 °C, 60 ± 5% relative humidity, and 16: 8 h L/D photocycle). Random samples of *D. citri* and alemow leaves were collected monthly and tested for ‘*Ca*. L. asiaticus’.

### Host preference evaluation and ‘*Ca*. L. asiaticus’ infection

The prepared trees were divided into two groups. One group was kept in a certified *D. citri*-free greenhouse to be used as healthy controls, while the other group was challenged with ‘*Ca*. L. asiaticus’-infected psyllids. To evaluate behavioral responses to sweet orange varieties, we followed the methodology outlined by Killiny et al. [22]). Briefly, one tree of each variety was randomly placed at the corners of 75 × 75 × 75 cm insect-proof cages (#1466CV, BioQuip Products, Rancho Dominguez, CA). Newly emerged *D. citri* adults (~ 3 d old) were collected from infected colonies without discrimination against age or gender for the trees’ exposure. In each replicate (cage), 100 adults of *D. citri* were collected into separate 50-mL vials and released in the center of each cage after removing the vial cap. The number of adults that settled on each plant was recorded daily for two weeks, and the experiments were conducted in eight replicates. After three months, both the infected and non-infected trees were transferred to a common evaluation *D. citri*-free greenhouse for an additional six months to assess the infection rate and the titer of the ‘*Ca*. L. asiaticus’.

### ‘*Ca*. L. asiaticus’ titer determination

Total DNAs from trees, 6- and 12-month ACP feeding, were isolated from the leaf petioles and midveins of fully expanded leaves using a GeneJET Plant Genomic DNA Purification kit (Thermo Fisher Scientific, Waltham, MA, USA). DNAs from psyllid were extracted using Quick-DNA Miniprep Plus (Zymo, Irvine, CA) according to the manufacturer’s instructions. The detection of ‘*Ca*. L. asiaticus’ DNA was performed through qPCR, employing TaqMan™ Master Mix and OI1/OI2 primers as described by Tatineni et al. [23] using the QuantStudio™ 3 System.

### Estimation of total phenolic and flavonoid content

The foliar phenolic compound content (TPC) was estimated using the Folin–Ciocalteu method with slight modifications [24]. The total flavonoid content in the leaf samples was estimated using a colorimetric assay with aluminum chloride [25]. For each treatment, 12 months biological replicates were used, with 12 months technical replicates per biological replicate.

### Hydrogen peroxide estimation and in situ histochemical localization of O_2_^•−^ and H₂O₂

To measure hydrogen peroxide (H₂O₂) levels, six leaves were collected, homogenized in 1 mL of 0.1% trichloroacetic acid (TCA) on ice, followed by centrifugation [26]. H₂O₂ content was quantified by mixing 100 µL of the supernatant with 100 µL of 10 mM potassium phosphate buffer and 200 µL of potassium iodide, then measuring absorbance at 390 nm. The H₂O₂ concentration was determined using a calibration curve from a standard H₂O₂ solution, with results expressed as nmol g⁻¹ FW according to Velikova et al. [27].

Histochemical staining techniques were used to localize superoxide (O_2_^•−^) and hydrogen peroxide (H₂O₂) in the leaves. For O_2_^•−^ detection, leaves were submerged in a 1 mg·mL^−1^ nitro blue tetrazolium (NBT) solution in a 50 mM potassium phosphate buffer (pH 6.4) followed by vacuum infiltration for 10 minutes. They were then incubated at room temperature until dark blue formazan precipitates appeared, signaling O_2_^•−^ accumulation according to Killiny et al. [28]. For H₂O₂ localization, leaves were vacuum-infiltrated with a 1 mg·mL^−1^ 3,3’-diaminobenzidine (DAB) solution in 10 mM MES buffer (pH 6.5) for 15 min, followed by 8 h of incubation in the dark. The leaves were bleached in boiling ethanol to highlight the blue or brown staining. Furthermore, ROS was detected by incubation in a 50 µM H2DCFDA-fluorescent probe for 1 h in a loading buffer (10 mM MES, 50 mM KCl, pH 7.2). The leaves were examined under a fluorescence microscope with a Rhodamine filter.

### In vivo collection and analysis of volatile organic compounds

Emitted volatile organic compounds (VOCs) were collected in vivo using static headspace solid phase microextraction (SPME) as described previously [22, 29]. A mixed polarity SPME fiber (57328U, Supelco, Bellefonte, PA, USA; needle size 24) was used with a manual holder (MilliporeSigma, Burlington, MA). This SPME fiber effectively traps a wide range of VOCs [30]. The collected VOCs were analyzed by gas chromatography-mass spectrometry (GC-MS) under conditions outlined in previous studies [22, 29].

### Starch content and callose deposition estimation

Leaves were bleached in boiling ethanol for 1 h to remove alcohol-soluble materials [31]. After bleaching, the leaves were rinsed with distilled water. Starch was visualized using iodine staining using Iodine-potassium iodide solution (2 g I_2_ and 20 g KI per liter) at room temperature for 1 h [28]. Starch content was measured following the method outlined by Rosales and Burns [32].

Pieces of stem bark (outer layer of the stem containing phloem tissue) were collected from trees and fixed in ECA solution (60% ethanol, 30% chloroform, 10% acetic acid) for 2 h and then were treated with 2 N NaOH for 5–10 min. After washing with 0.1 M K_2_HPO_4_, pH 8.5, the tissues were incubated with 0.05% aniline blue [33, 34]. The RFU of the aniline blue stain was measured using ZEISS ZEN 3.1 blue edition image processing software.

### Polar metabolites estimation

The polar metabolites in the leaf samples were converted to their volatile trimethylsilyl (TMS) ethers by two-step derivatization with methoxamine hydrochloride and N-methyl-N-trimethylsilyl trifluoroacetamide as previously detailed [18]. Derivatized samples were analyzed using a nonpolar ZB-5MS column (Phenomenex, Torrence, CA, USA) in electron impact (EI) mode, with ultrapure hydrogen as the carrier gas at a flow rate of 1.0 mL·min^−1^, following the same temperature programming conditions previously described by Killiny [35]. Total ion chromatograms were processed using TurboMass software (v. 5.4.2, PerkinElmer, Shelton, CT). Compounds of interest were identified by comparing the experimental mass spectra with Library entries from the Wiley 9th edition and NIST 2011 mass spectral libraries and using authentic standards. Chromatographic peak areas were normalized to the area of the internal standard, ribitol.

### RNA isolation, cDNA library construction, and RNA-sequencing

Total RNA was extracted from one-year-old growing trees in the greenhouse (three varieties, each with three biological replicates derived from independent trees) using TRIzol^®^ and the Direct-zol RNA Miniprep Plus Kit, following the manufacturer’s protocol. The RNA quality and quantity were analyzed using a NanoDrop™ One/OneC Microvolume UV-Vis Spectrophotometer (Thermo Scientific). High-quality RNA samples (RNA integrity number (RIN) > 6.5) were sent for RNA sequencing (RNA-seq). For cDNA Library preparation, 1 µg of high-quality total RNA was used per sample. mRNA was enriched using oligo(dT) beads to capture polyadenylated transcripts, followed by fragmentation. First-strand cDNA synthesis was performed using random hexamer primers and reverse transcriptase, and second-strand cDNA was synthesized to produce double-stranded cDNA. After end-repair, A-tailing, and adapter ligation, libraries were amplified by PCR and purified. Library quality and quantity were assessed using standard QC protocols before sequencing. RNA quality validation, cDNA library construction, and high-throughput sequencing were performed at the Interdisciplinary Center for Biotechnology Research (ICBR) NextGen DNA Sequencing and Gene Expression Cores, University of Florida. Sequencing was conducted on the Illumina NovaSeq X platform, generating 150 bp paired-end reads with an average output of more than 20 million reads per sample.

Reads were cleaned up with the cutadapt program [36] to trim off sequencing adaptors and low-quality bases with a quality phred-like score < 20. Reads < 40 bases were excluded from RNA-Seq analysis. The genome of *Citrus sinensis* (*Citrus sinensis* v1.0 (NCBI)) from the Citrus Pan-genome to Breeding Database (CPBD) was used as the reference sequence for RNA-seq analysis [37]. The cleaned reads of each sample were mapped individually to the reference sequences using the read mapper of the STAR package (Spliced Transcripts Alignment to a Reference, v2.7.9a) [37]. The mapping results were processed with the HTSeq (High-Throughput Sequence Analysis in Python, v0.11.2) [38], samtools, and scripts developed in-house at ICBR of UF to remove potential PCR duplicates, choose and count uniquely mapped reads for gene expression analysis. PCA analysis (for detecting outlier samples) and volcano plot analysis based on all identified genes in each analysis were performed with the R-package (v4.4). The counted reads of each gene were analyzed by a DESeq2-based R pipeline. Significant up-and down-regulated genes were selected using the *padj-*value and log2 fold-change for downstream analysis. Volcano plots and heatmaps with 2 < log_2_FoldChange <−2 and *padj-value ≤* 0.05 were generated to visualize differential gene expression with in-house R scripts. Pathway enrichment analyses were achieved using GSEA (Gene Set Enrichment Analysis) [39]. The log2 fold-change between the genotypes was ranked and run through the GSEA using the clusterProfiler (v3.16.1) package in R (v.4.4) [40]. Pathway analysis was performed using the pathways function of MapMan (MapMan version 3.5.0).

### Gene expression analysis for RNA-seq validation

RT-qPCR for gene expression analysis was performed using SYBR^®^ Green PowerUp™ PCR Master Mix, gene-specific primers (Integrated DNA Technologies, Inc., Coralville, IA, USA), and the QuantStudio™ 3 System (Thermo Fisher Scientific, Massachusetts, USA). The relative mRNA levels were compared to those of the endogenous *CsActin* gene using the same 2^−ΔΔCT^ method [41]. Twelve genes were selected to evaluate the reliability of the transcriptome data using the same sequenced samples (Supplementary Table 1).

### Statistical analysis

Statistical analyses were performed using JMP Pro v17 software. The three varieties were compared under two conditions (healthy and *‘Ca.* L. asiaticus’-infection) using analysis of variance (ANOVA) followed by the post hoc Tukey–Kramer honestly significant difference (HSD). For each variety, a two-tailed *t*-test was used for pairwise statistical comparison between the healthy and *‘Ca.* L. asiaticus’-infected. Data on ACP landing (%) were analyzed using both linear mixed-effects models (LMMs.) and non-parametric tests to account for data characteristics and experimental design. A linear mixed-effects model was fitted with variety as a fixed effect and cage (eight replicates) as a random effect to account for block structure. Model assumptions were assessed, and due to some variance heterogeneity and borderline normality, a complementary non-parametric approach was applied using the Kruskal-Wallis test followed by Dunn’s post-hoc test with Benjamini-Hochberg adjustment for multiple comparisons. Estimated marginal means and standard errors from the mixed model were calculated for visualization and interpretation. All analyses were conducted using R 4.4.3 with packages lme4, lmerTest, emmeans, and FSA. The two-way hierarchical cluster analysis (HCA) associated with heatmaps was performed using average values. Distance and linkage of HCA were conducted using Ward’s minimum variance method [42], with 95% confidence between groups from the discriminant function analysis to construct the similarity dendrograms. Statistical significance was established at α = 0.05.

## Results

### ‘Parson Brown’ showed decreased *D. citri* attraction and landing

**Fig. 1:**
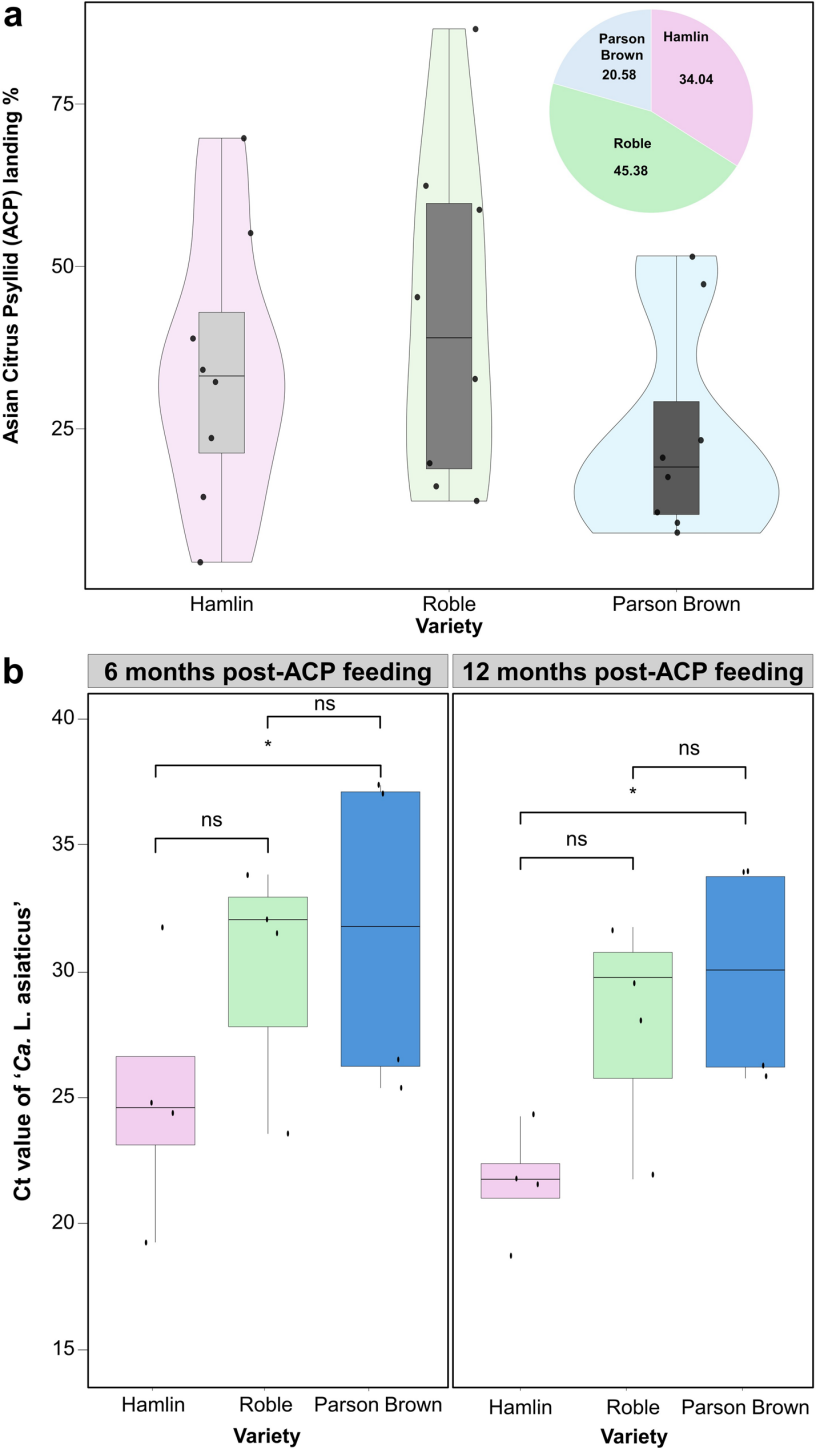
**a** Bar chart depicting the ratio of Asian citrus psyllid (ACP) landing on the three varieties from seven independent experiments and pie chart illustrating the percentage of ACP landing and settling on ‘Hamlin,’ ‘Roble’, and ‘Parson Brown’ (*n* = 8). **b** Quantitative PCR (qPCR) analysis showing cultivar-specific differences in ‘*Ca*. L. asiaticus’ titer (Ct) 6 and 12 months post-ACP feeding. Data are presented as mean ± standard deviation, *n* = 4. The statistical significance was established at *p* < 0.05

Following the release of *D. citri* adults, psyllids landed on all tested sweet orange varieties (‘Parson Brown’, ‘Hamlin’, and ‘Roble’) (Fig. 1a). Among these, ‘Parson Brown’ exhibited a lower average landing percentage (20.58%) compared to ‘Hamlin’ (34.38%) and ‘Roble’ (45.04%). However, the linear mixed-effects model, which accounted for cage as a random effect, showed no statistically significant differences in ACP landing percentages among the varieties (Variety: F (2,18) = 0.94, *p* = 0.41). Complementary non-parametric analysis using the Kruskal-Wallis test supported these results (χ² = 2.23, df = 2, *p* = 0.33), and Dunn’s post-hoc test with Benjamini-Hochberg adjustment indicated no statistically significant pairwise differences.

### Parson Brown’ harbors less titer of ‘*Ca*. l. asiaticus

Quantitative PCR was conducted at both 6 and 12 months post-inoculation with ‘*Ca*. L. asiaticus’ to assess bacterial titer in leaf petioles across the three citrus varieties. A significant difference in infection levels was recorded when comparing the mean cycle threshold (Ct) values of ‘Hamlin’ and ‘Parson Brown’ (*p* = 0.034), recording mean Ct values ± standard deviation for ‘*Ca.* L. asiaticus’ of 25 ± 2 for ‘Hamlin’, and 32 ± 3 for ‘Parson Brown’ (Fig. 1b). A similar trend was observed at 12 months post-inoculation, where ‘Parson Brown’ continued to exhibit higher Ct values compared to ‘Hamlin’. However, no significant differences were observed when comparing ‘Roble’ with either ‘Hamlin’ or ‘Parson Brown’ at either time point.

### RNA-Seq metrics for sweet orange varieties under healthy and infected trees

A total of 18 RNA samples (three replicates from each variety: ‘Hamlin’ (H), ‘Roble’ (R), and ‘Parson Brown’ (PB)) under two conditions (healthy and ‘*Ca*. L. asiaticus’-infected) were sequenced, generating an average of 26.52 million raw reads per sample, with a range from 16.95 to 32.15 million reads. After quality filtering, clean reads were aligned to the *Citrus sinensis* v1.0 reference genome, with mapping rates ranging from 90.01 to 93.02%, and fewer than 3.60% unmapped reads across all libraries (Supplementary Table 2). The expression of twelve conserved genes was assayed through RT-qPCR. The RT-qPCR results were consistent with the RNA-seq data, and the mRNA expression of these genes was either significantly up- or down-regulated in the tested samples (Supplementary Fig. 1 and Supplementary Table 1).

### ‘*Ca*. L. asiaticus’-infection alters the transcriptome profile in sweet oranges

**Fig. 2:**
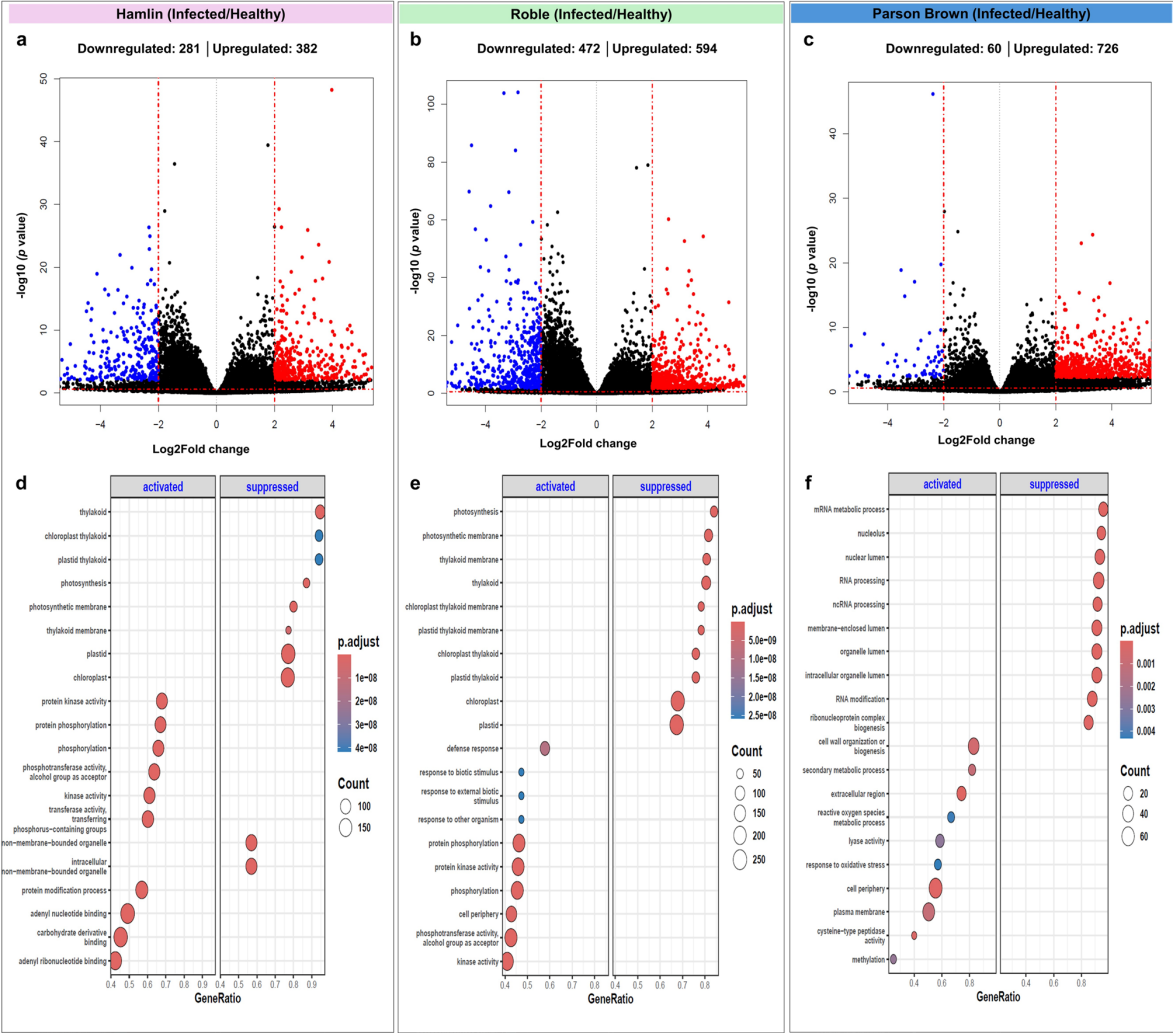
Differentially expressed genes (DEGs) and their functional enrichment across three sweet orange varieties (‘Hamlin’, ‘Parson Brown’, and ‘Roble’) under controlled conditions compared to the infected trees. Panels **a**, **b**, and **c** show a volcano plot and the number of DEGs in the comparisons. Panels **d**, **e**, and **f** illustrate gene set enrichment (GSE) analysis

**Fig. 3:**
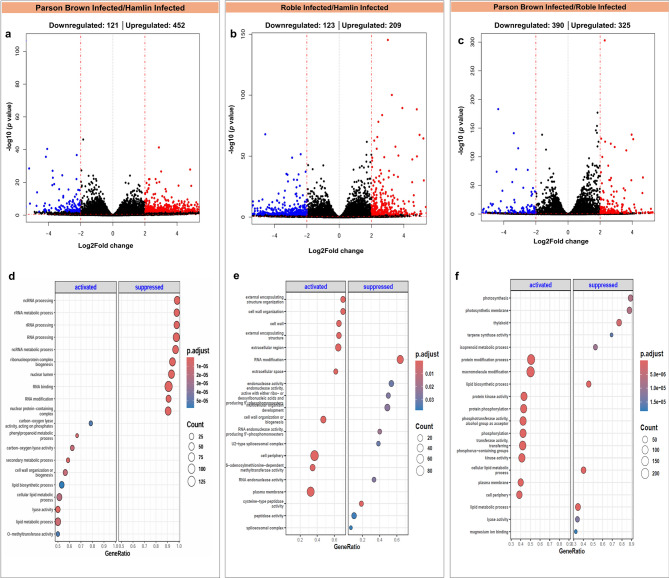
Differentially expressed genes (DEGs) and their functional enrichment across three sweet orange varieties (‘Hamlin’, ‘Parson Brown’, and ‘Roble’) following ‘*Ca*. L. asiaticus’ -infection compared to each other. Panels **a**, **b**, and **c** show volcano plot and the number of DEGs in the comparisons. Panels **d**, **e**, and **f** illustrate gene set enrichment (GSE) analysis

Upon infection, we observed significant alterations in differentially expressed genes (DEGs), with 663, 1,066, and 786 upregulated DEGs in ‘Hamlin’, ‘Roble’, and ‘Parson Brown’, respectively (Fig. 2). Additionally, a comparative analysis among the infected trees revealed 573 differentially expressed genes (DEGs) between infected ‘Hamlin’ and infected ‘Parson Brown’, 332 DEGs between infected ‘Hamlin’ and infected ‘Roble’, and 715 DEGs between infected ‘Parson Brown’ and infected ‘Roble’ (Fig. 3).

### Functional classification by GO analysis and KEGG pathway mapping

We observed enrichment of various Gene Ontology (GO) categories upon infection in ‘Hamlin,’ ‘Roble,’ and ‘Parson Brown’ trees (Figs. 2 and 3, Supplementary Fig. 3, 4). For ‘Hamlin’ trees, the significant enrichment of processes is related to aminoglycan, chitin metabolism, and cell wall biogenesis. Additionally, processes linked to biotic stress responses, such as fungal defense, heat stress, and the cell surface receptor signaling pathway, were enriched. On the other hand, ‘Roble’ trees demonstrated an enrichment in polysaccharide metabolism and lignin biosynthesis, water transport, and glycosyltransferase and oxidoreductase activities. ‘Parson Brown’ exhibited enrichment in secondary metabolic processes, including the terpenoid biosynthetic process (GO:0006721) and flavonoid biosynthesis pathways (GO:0009813), along with pectin catabolism and cell wall biogenesis and organization (GO:0071554, GO:0071669, GO:0009505), which was not as prominently enriched in the other two varieties. Additionally, chloroplast thylakoid membranes (GO:0009579, GO:0042651) were enriched in ‘Parson Brown’.

### *Ca*. L. asiaticus’-infection altered phenylpropanoid pathway in ‘Parson Brown

**Fig. 4:**
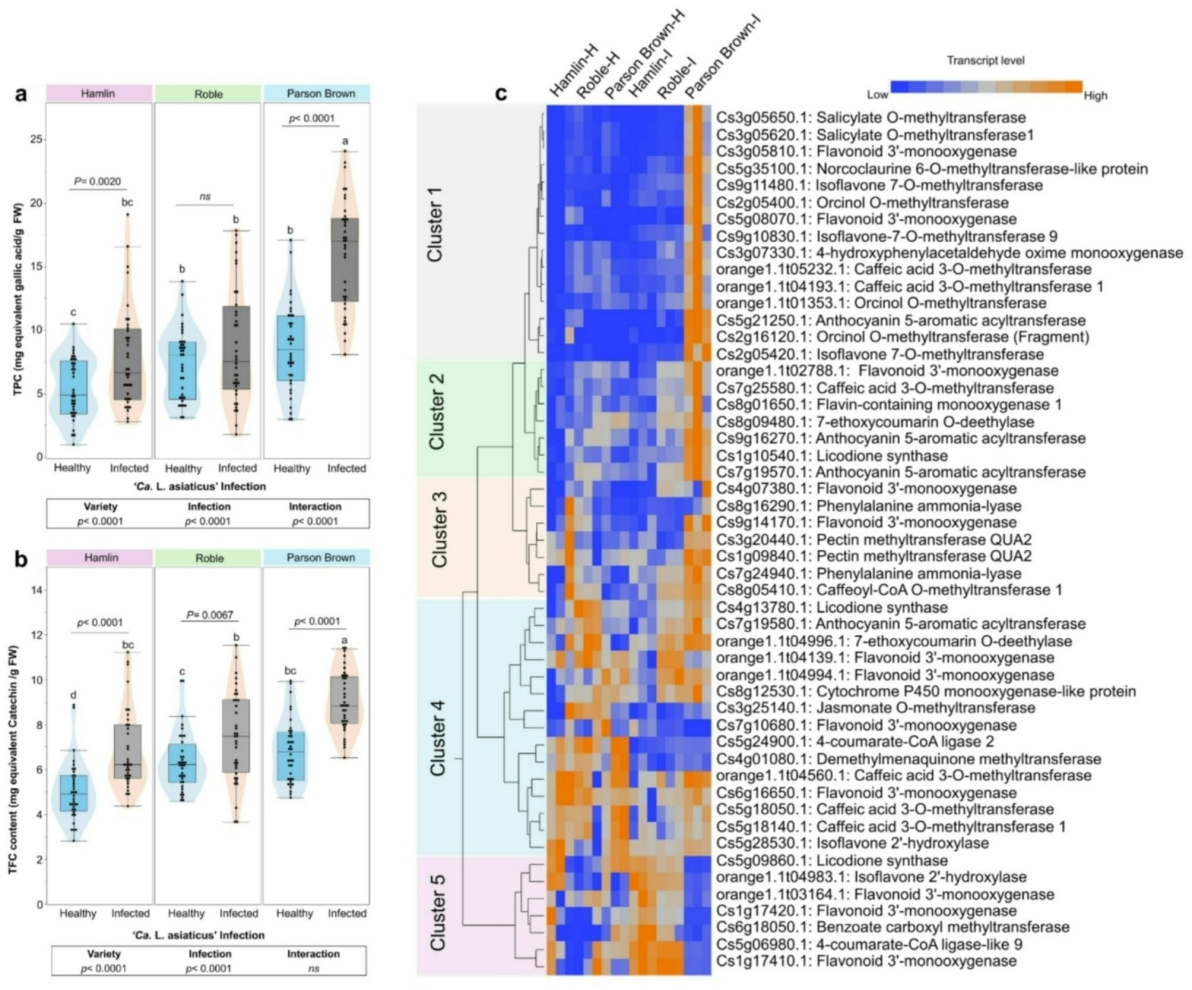
Overview of metabolite content and phytohormone-related gene expression profiles in sweet orange varieties under healthy and infected conditions. **a** Total Phenolic Content (TPC) and **b** Total Flavonoid Content (TFC) in Sweet Orange varieties under Healthy and Infected Conditions. Boxplots show the interquartile ranges (25th to 75th percentiles of the data), and black dots represent the raw data (*n* = 30). Two-way ANOVA was performed to assess the effects of variety, treatment (healthy vs. infected), and their interaction. Additionally, paired t-tests were conducted to compare healthy and infected samples within each variety. Significant differences between groups are indicated by different letters; groups that share any letter are not significantly different (*p* < 0.05). **c** Gene expression profiles of metabolite- and phytohormone-related genes. One-way hierarchical cluster analysis (HCA) with heatmaps was performed using transcript levels of genes involved in metabolite biosynthesis and phytohormone signaling pathways. The cells represent the reads of each gene (*n* = 3)

The levels of total phenolic compounds (TPC) and total flavonoid content (TFC), and their upstream regulatory genes, were recorded across the varieties under both healthy and infection conditions. In terms of TPC, infected ‘Parson Brown’ exhibited the highest content (16.18 ± 4.25 mg·g^−1^ FW), followed by infected ‘Roble’ at 8.72 ± 4.56 mg·g^−1^ FW, and infected ‘Hamlin’ at 7.83 ± 3.96 mg·g^−1^ FW. Healthy ‘Parson Brown’ had a TPC of 8.63 ± 3.43 mg·g^−1^ FW, while ‘Roble’ and ‘Hamlin’ had 7.38 ± 2.73 and 5.37 ± 2.34 mg·g^−1^ FW, respectively (Fig. 4a). For TFC, infected ‘Parson Brown’ also showed the highest level (9.09 ± 1.30 TFC content (mg·g^−1^ FW), followed by infected ‘Roble’ at 7.46 ± 2.01 mg·g^−1^ FW, and infected ‘Hamlin’ at 6.85 ± 1.76 mg·g^−1^ FW (Fig. 4b).

Forty-two genes associated with the phenylpropanoid pathway were identified in our RNA-seq dataset. Of them, Two phenylalanine ammonia-lyase genes, which catalyze the first committed step converting phenylalanine to cinnamic acid and initiate the biosynthesis of phenolic compounds, along with caffeic acid 3-O-methyltransferase and caffeoyl-CoA O-methyltransferase, enzymes involved in the methylation and modification of lignin precursors and other phenolics, were significantly expressed in infected ‘Parson Brown’ (Fig. 4c). Flavonoid biosynthesis-related genes, including flavonoid 3’-monooxygenase and anthocyanin 5-aromatic acyltransferase, which contribute to the production of flavonoids and anthocyanins with antioxidant and antimicrobial properties, were significantly expressed in ‘Parson Brown’. Genes involved in methylation processes, such as pectin methyltransferase, salicylate O-methyltransferase, and jasmonate O-methyltransferase, were also prominently upregulated in ‘Parson Brown’ compared to other varieties; these enzymes modify cell wall components and plant hormones, influencing defense signaling and structural integrity. Isoflavonoid biosynthetic genes, such as isoflavone 7-O-methyltransferase (Cs9g11480.1, Cs9g10830.1 and Cs2g05420.1), which are involved in synthesizing compounds linked to pathogen resistance and stress tolerance, were also upregulated (Fig. 4c). Among the differentially expressed genes, salicylate O-methyltransferase (S-adenosyl-L-methionine-dependent methyltransferases: Cs3g05650.1 and Cs3g05620.1) was the most significantly upregulated in infected ‘Parson Brown’ leaves. This enzyme plays a critical role in the salicylic acid (SA) pathway by converting SA into methyl salicylate, a key mobile signal involved in systemic acquired resistance.

### ‘*Ca*. L. asiaticus’-infection induces oxidative stress responses

**Fig. 5:**
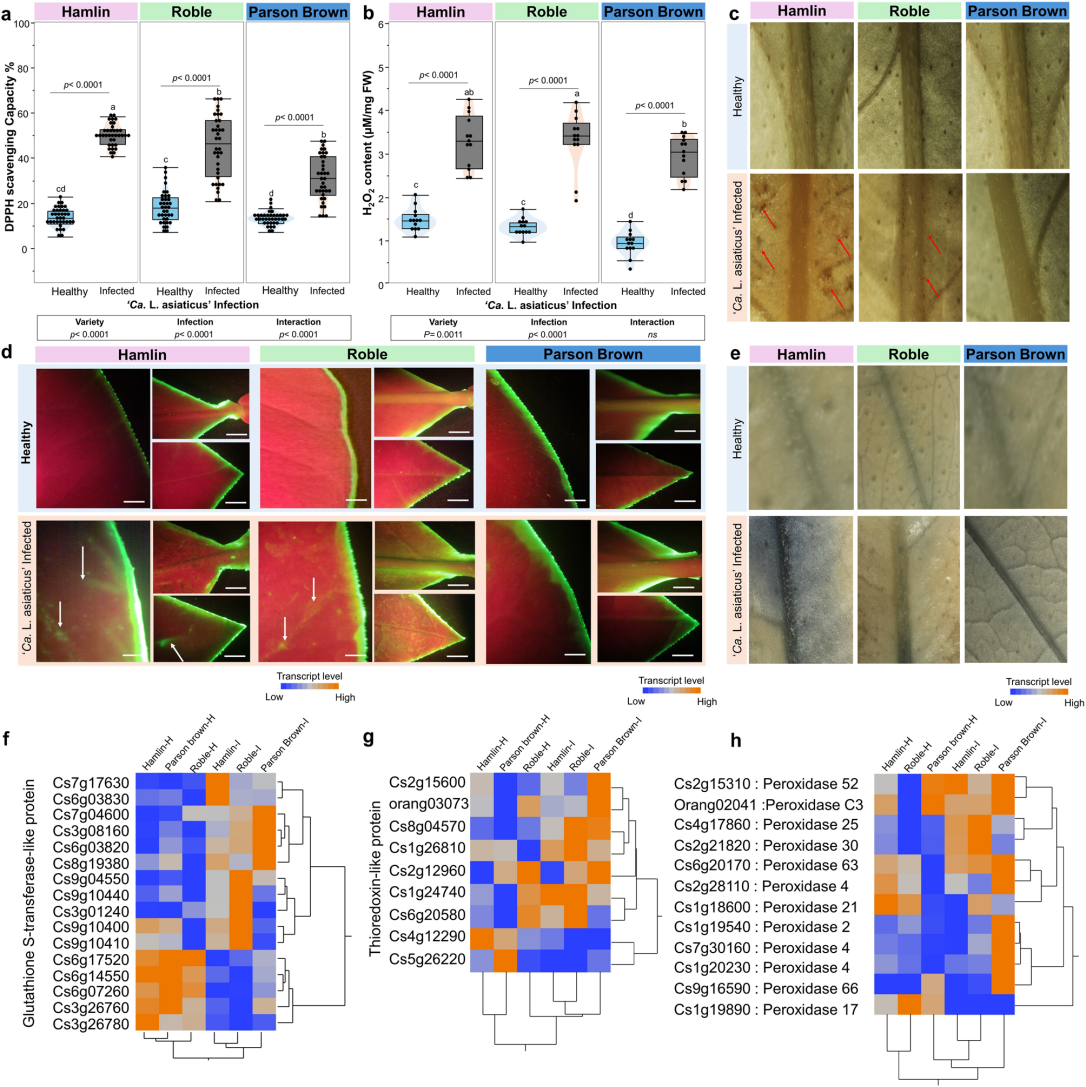
Antioxidant Activity and Gene Expression Profiles in Sweet Orange Cultivars under Infection. **a** DPPH scavenging capacity (%). **b** Hydrogen peroxide (H_2_O_2_) levels (µM·mg^−1^). **c** In situ histochemical analysis of H_2_O_2_ using DAB staining. c In situ histochemical detection of hydrogen peroxide (H₂O₂) localization using DAB staining in citrus leaves. The red arrows are pointing to areas of brown precipitate, which indicates the presence of hydrogen peroxide (H₂O₂). **d** ROS production levels as indicated by H2DCFDA staining. **e** In situ staining for superoxide anion (O_2_^•−^) using NBT. The white arrows are pointing to areas of ROS fluorescence. Boxplots show the interquartile ranges (25th to 75th percentiles of the data), and black dots represent the raw data (*n* = 30). Two-way ANOVA was performed to assess the effects of variety, treatment (healthy vs. infected), and their interaction. Additionally, paired t-tests were conducted to compare healthy and infected samples within each variety. Significant differences between groups are indicated by different letters; groups that share any letter are not significantly different (*p* < 0.05). Two-way hierarchical cluster analysis and heatmap of the glutathione S-transferase-like proteins **f**, Thioredoxin-like protein-related genes **g**, and peroxidase genes **h**. Two-way hierarchical cluster analysis (HCA) with heatmaps was performed using transcript levels of genes involved in antioxidant-related genes. The cells represent the reads of each gene (*n* = 3)

We observed significant differences in DPPH scavenging capacity and hydrogen peroxide (H_2_O_2_) levels among the three varieties. Infected ‘Parson Brown’ trees exhibited the lowest DPPH scavenging capacity (30.59% ± 9.91), followed by ‘Roble’ (44.91% ± 13.87) and ‘Hamlin’ (48.64% ± 9.96) (Fig. 5a). Similarly, H_2_O_2_ levels were lowest in infected ‘Parson Brown’ trees (2.95 ± 0.45 µM·mg^−1^), while ‘Roble’ (3.32 ± 0.64 µM·mg^−1^) and ‘Hamlin’ (3.29 ± 0.61 µM·mg^−1^) had higher levels (Fig. 5b). In situ histochemical analysis of H_2_O_2_ further corroborated these results. ‘Hamlin’ leaves showed darker brown staining upon DAB-based localization recording integrated optical density (IOD) of 0.28 ± 0.01 compared to ‘Parson Brown’ (0.19 ± 0.01 IOD) and ‘Roble’ (0.26 ± 0.01 IOD) (Supplementary Fig. 5a). Additionally, in situ staining for superoxide anion (O^2−^) using NBT revealed more intense blue coloration in ‘Hamlin’ leaves than in ‘Parson Brown’ and ‘Roble’ (Fig. 5e, Supplementary Fig. 5b). Moreover, H2DCFDA staining showed that ‘Hamlin’ leaves exhibited higher green fluorescence than ‘Roble and ‘Parson Brown’ (Fig. 5d, Supplementary Fig. 5c).

The expression of glutathione S-transferase-like genes (GSTs), which play key roles in detoxification and antioxidant defense, varied significantly among the three citrus varieties in response to *‘Ca*. L. asiaticus’ infection. Infected ‘Parson Brown’ exhibited higher expression of specific GST homologs, including Cs7g04600, Cs3g08160, Cs6g03820, and Cs8g19380, compared to both ‘Hamlin’ and ‘Roble’. In contrast, only Cs7g17630 and Cs6g03830 were significantly upregulated in ‘Hamlin’. ‘Roble’ showed elevated expression of several GSTs located on chromosome 9, namely Cs9g04550, Cs9g10400, Cs9g10410, Cs9g10440, in addition to Cs3g01240 (Fig. 5f). A similar pattern was observed for thioredoxin-like protein-related genes, which are involved in maintaining cellular redox balance. While Cs1g24740 and Cs2g12960 were upregulated in both ‘Hamlin’ and ‘Roble’, ‘Parson Brown’ specifically showed increased expression of Cs2g15600, orange1.1t03073, and Cs2g12960 (Fig. 5g), suggesting a distinct antioxidant regulatory network. Expression of peroxidase genes also displayed cultivar-specific trends. Infected ‘Parson Brown’ showed strong upregulation of several peroxidases, including orange1.1t02041 (Peroxidase C3), Cs6g20170 (Peroxidase 63), Cs2g28110, Cs1g19540, Cs7g30160, Cs1g20230 (all annotated as Peroxidase 4), and Cs9g16590.1 (Peroxidase 66) (Fig. 5h). In contrast, Peroxidase 25 and Peroxidase 30 were more highly expressed in ‘Roble’, while Peroxidase 52 was strongly expressed in both infected ‘Hamlin’ and ‘Parson Brown’.

### *Ca*. L. asiaticus’-infection altered terpenoid biosynthesis in ‘Parson Brown

**Fig. 6:**
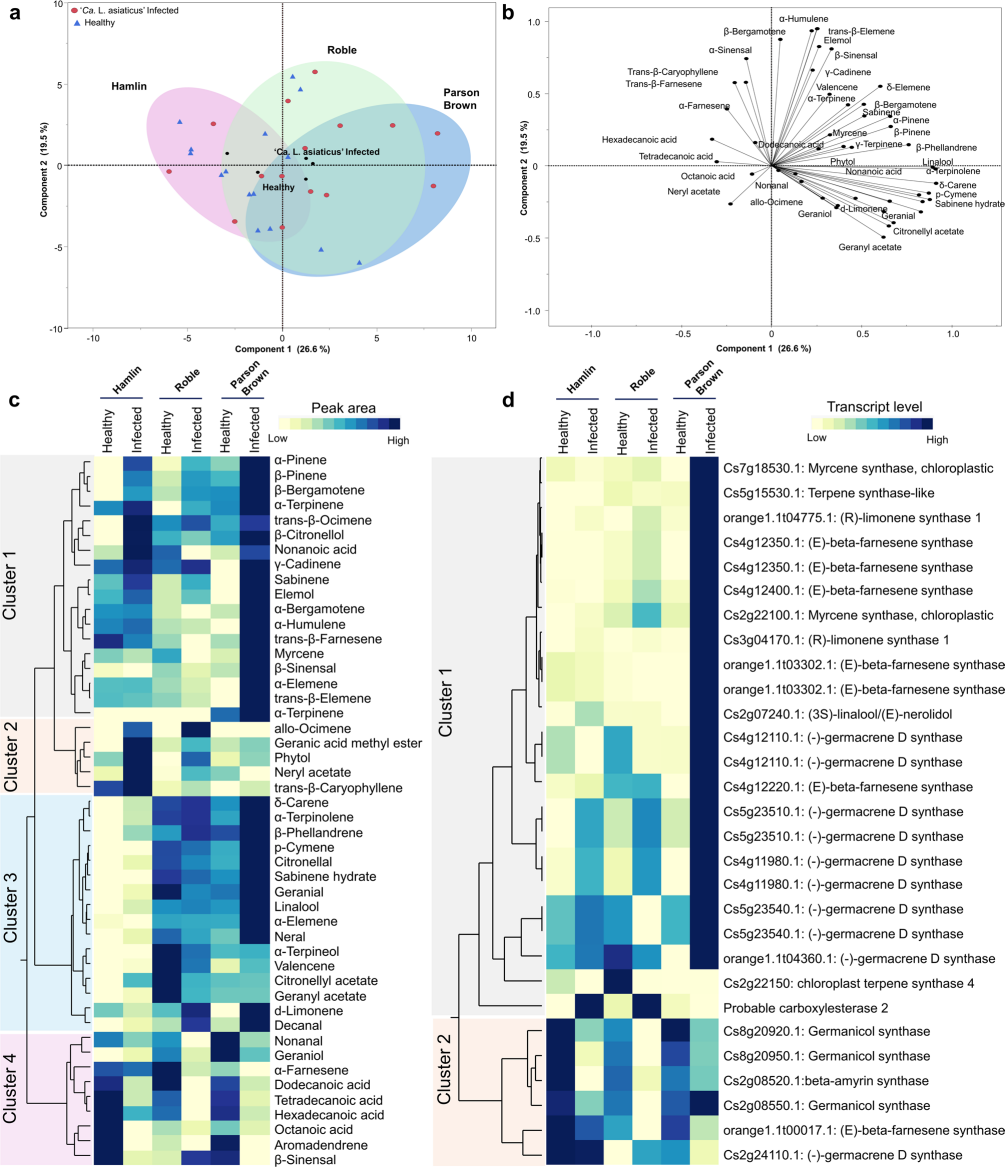
Volatile organic compounds (VOCs) detected from sweet orange variety leaves under healthy and ‘*Ca.* L. asiaticus’- infection. **a**,** b** Principal component analysis (PCA) shows the multivariate variation among the VOCs released from the leaves of the two conditions. **a** PCA scatter plot. **b** PCA loading plot. Colored symbols correspond to the VOCs released from the treatments for the three varieties, both healthy and infected. **c** one-way hierarchical cluster analysis and heatmap of the VOCs released from the three varieties. Rows represent compounds, and columns represent the two treatments for the three varieties. The cells represent the mean peak area of each compound (*n* = 5). **d** One-way hierarchical cluster analysis and heatmap of the terpene-related DEGs expressed in the three varieties. The cells represent the reads of each gene (*n* = 3)

Principal Component Analysis (PCA) analysis revealed distinct volatile profiles among varieties, with clear differentiation based on VOCs under both healthy and infected conditions (Fig. 6a, b). The first principal components (PC1 and PC2) collectively represented 46.1% of the total variance, with PC1 contributing 26.6% and PC2 19.5%.

A heatmap using the averages of detected volatiles (Fig. 6c) identified four VOC clusters. Cluster 1 represented VOCs released in higher concentrations by ‘Parson Brown’ compared to the other varieties, including α-pinene, β-pinene, β-bergamotene, α-terpinene, *trans*-β-ocimene, β-citronellol, nonanoic acid, γ-cadinene, sabinene, elemol, α-bergamotene, α-humulene, *trans*-β-farnesene, β-sinensal, and myrcene. Cluster 2 indicated VOCs released from infected ‘Hamlin’, primarily consisting of allo-ocimene, geranic acid methyl ester, phytol, neryl acetate, and *trans*-β-caryophyllene. Cluster 3 highlighted compounds more abundant in ‘Parson Brown’ and ‘Roble’, in both healthy and infected statuses, compared to ‘Hamlin’, including δ-carene, α-terpinolene, β-phellandrene, p-cymene, citronellal, citronellal, sabinene hydrate, geranial, linalool, α-elemene, and neral. Cluster 4 revealed VOCs released in higher quantities from healthy control trees than those released in the infected trees. A two-tailed *t*-test comparing individual VOCs between infected and healthy control plants indicated that the VOC profile was significantly altered by infection (Supplementary Table 3).

Furthermore, differential expression analysis revealed upregulation of key enzymes involved in terpene biosynthesis in both healthy and infected trees. Cluster 1 showed elevated expression of multiple terpene biosynthetic genes specifically in infected *‘Parson Brown’*, including myrcene synthase, terpene synthase-like enzymes, (R)-limonene synthase 1, (E)-β-farnesene synthase, (3 S)-linalool/(E)-nerolidol synthase, (-)-germacrene D synthase, carboxylesterase 2, germanicol synthase, and β-amyrin synthase. These enzymes catalyze the production of diverse terpenoids that contribute to plant defense, signaling, and stress tolerance. In contrast, Cluster 2 was characterized by higher expression of terpene-related enzymes in healthy control trees across all three varieties, including germanicol synthase, β-amyrin synthase, (E)-β-farnesene synthase, and (-)-germacrene D synthase (Fig. 6d), suggesting that terpene metabolism is dynamically regulated in response to infection.

### ‘Parson Brown’ exhibits decreased callose deposition and starch accumulation

**Fig. 7:**
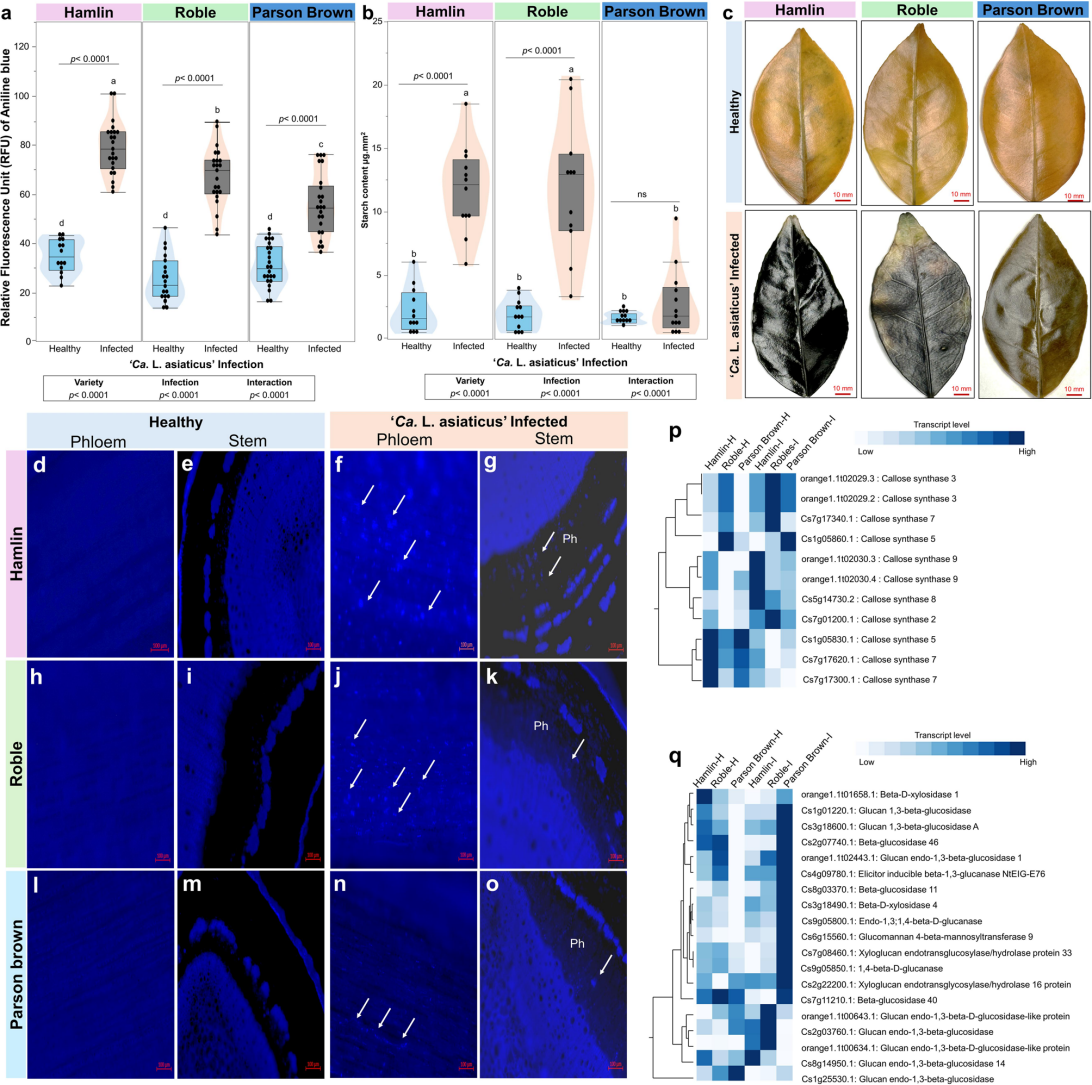
Callose accumulation, starch content, and gene expression in sweet orange variety leaves under healthy and ‘*Ca.* L. asiaticus’-infection. **a** Relative Fluorescence Unit (RFU) values from aniline blue staining of callose plugs in the stem phloem of ‘Hamlin,’ ‘Roble,’ and ‘Parson Brown.’ **b** Endogenous starch levels (µg·mg^−1^). Boxplots show the interquartile ranges (25th to 75th percentiles of the data), and white dots represent the raw data (*n* = 30). Two-way ANOVA was performed to assess the effects of variety, treatment (healthy vs. infected), and their interaction. Additionally, paired t-tests were conducted to compare healthy and infected samples within each variety. Different letters indicate statistically significant differences (*p* < 0.05). **c** In situ iodine staining images of starch accumulation. **d**,** h**,** i** callose staining using aniline blue of phloem tissues for the three varieties under control conditions, **f**,** j**,** n** callose staining of phloem tissues under ‘*Ca.* L. asiaticus’- infection. **e**,** j**,** m** callose staining of stem tissues for the three varieties under control conditions, **g**,** k**,** o** callose staining of stem tissues under ‘*Ca.* L. asiaticus’- infection. The white arrows are pointing to areas of callose deposition. **p** One-way hierarchical cluster analysis and heatmap of expression analysis of callose synthase genes, and **q** glucosidase-related genes. The cells represent the reads of each gene (*n* = 3)

Analysis of the Relative Fluorescence Unit (RFU) using Aniline Blue staining revealed significant differences in the percentage of callose plugs in the stem phloem of ‘*Ca.* L. asiaticus’-infected trees (Fig. 7a, e-k). The RFU values were 78.57 ± 10.89 in ‘Hamlin’, 67.05 ± 11.92 in ‘Roble’, and 55.72 ± 12.67 in ‘Parson Brown’ (Fig. 7a). Additionally, we observed a significant reduction in endogenous starch levels in ‘Parson Brown’ leaves, recording 2.19 ± 1.76 µg·mm^−2^, compared to ‘Hamlin’ (11.72 ± 3.40 µg·mm^−2^) and ‘Roble’ (11.18 ± 5.34 µg·mm^−2^) (Fig. 7b). To confirm these findings, starch accumulation was localized in situ using the iodine staining technique after ethanol bleaching of leaves (Fig. 7c, d). Iodine staining revealed a dark blue coloration in ‘Hamlin’ and ‘Roble’ leaves, whereas ‘Parson Brown’ leaves exhibited a much lighter coloration. These visual results align with the findings from the colorimetric assays.

Callose synthases catalyze the synthesis of callose. This β−1,3-glucan polymer is deposited at sites of pathogen attack to reinforce cell walls and limit pathogen spread. These glucanases are involved in the degradation of β-glucans, which can Function in remodeling the cell wall and potentially in defense by degrading pathogen-derived glucans. Upon infection, key genes such as callose synthase 5, callose synthase 7, and callose synthase 3 exhibited significant upregulation, with the highest expression observed in ‘Hamlin’, followed by ‘Roble’. At the same time, the ‘Parson Brown’ displayed lower expression levels (Fig. 7p). Furthermore, glucan- and glucosidase-related genes include glucan endo-1,3-beta-glucosidase, Glucan 1,3-beta-glucosidase A exhibited higher expression in ‘Parson Brown’ compared to ‘Hamlin’ and ‘Roble’ (Fig. 7q).

### *Ca*. L. asiaticus’-infection alters disaccharide and sugar alcohols in ‘Parson Brown

**Fig. 8:**
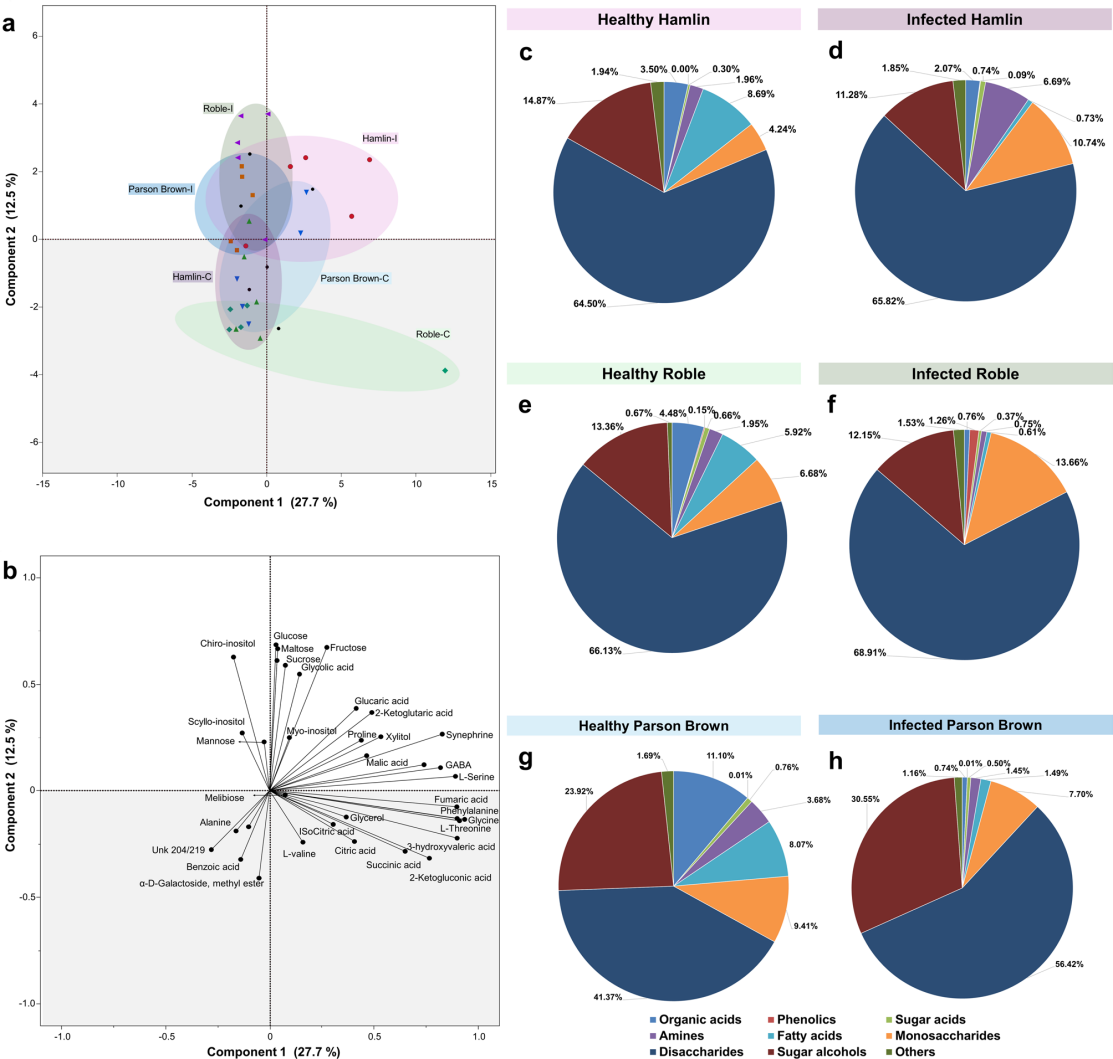
Metabolite Profiling of Healthy and ‘*Ca.* L. asiaticus’ -Infected sweet orange trees ‘Hamlin’, ‘Roble’, and ‘Parson Brown’. **a**,** b** Principal component analysis (PCA) shows the multivariate variation among the metabolites accumulated in the leaves of the two conditions. **a** PCA scatter plot. **b** PCA loading plot. Colored symbols correspond to the metabolites in the three varieties, both healthy and infected. **c-h** Percentage composition of metabolite groups in the leaves of healthy and infected sweet orange varieties (‘Hamlin’, ‘Parson Brown’, and ‘Roble’)

Among the 51 metabolites identified, 36 showed no significant differences between healthy and infected trees (Fig. 8, Supplementary Table 4). Infected ‘Hamlin’ showed elevated levels of pyruvic acid, L-threonine, synephrine, xylitol, galacturonic acid, and an unidentified galactoside, while infected ‘Roble’ had the highest levels of quinic acid, glucose, glucitol, and sucrose. Infected ‘Parson Brown’ trees had the highest levels of *chiro*-inositol and *scyllo*-inositol. A concurrent reduction in *trans*-α-octadecenoic acid was found across all infected trees (Supplementary Table 4). Comparing the overall content of each category revealed higher levels of monosaccharides in ‘Hamlin’ and ‘Roble’ compared to ‘Parson Brown’. All three varieties displayed a consistent reduction in fatty acid content upon infection. In healthy ‘Parson Brown’, disaccharides were the dominant metabolites (41.37%), followed by sugar alcohols (23.92%) and organic acids (11.10%) whereas, the infected leaves of ‘Parson Brown’ exhibited significant increase in disaccharides (56.42%) and sugar alcohols (30.55%) but a significant decrease in monosaccharides, organic acids, and amine metabolites (Fig. 8).

## Discussion

‘Parson Brown’ sweet orange has been identified as a potentially HLB-tolerant cultivar compared to other commonly susceptible varieties under similar disease pressure [10]. In this study, we recorded reduced psyllid landing and lower ‘*Ca*. L. asiaticus’ titer in the ‘Parson Brown’. Subsequently, we integrated metabolomic and transcriptomic approaches to elucidate the tolerance mechanisms of ‘Parson Brown’ under endemic HLB conditions. This cultivar was compared to two other early sweet orange varieties, ‘Roble’ and ‘Hamlin’, which exhibit varying degrees of susceptibility to HLB. Our findings emphasize the complexity of tolerance in ‘Parson Brown’, which is driven by a combination of host-specific biochemical responses and interactions with both the psyllid vector and ‘*Ca*. L. asiaticus’ bacterium.

A key factor contributing to the tolerance of ‘*Ca.* L. asiaticus’ in ‘Parson Brown’ is the activation of the production of secondary metabolites such as phenolic compounds and flavonoids. These compounds play fundamental roles in plant defense mechanisms by exhibiting both antimicrobial and antioxidant properties, thereby reducing the toxic effects of ROS [43–45]. Phenylalanine ammonia-lyase (PAL) is a pivotal enzyme catalyzing the first step in the phenylpropanoid biosynthetic pathway, leading to the production of various secondary metabolites integral to plant defense. Daurelio et al. [46] conducted an in-depth transcriptomic analysis of *Citrus sinensis* nonhost interactions with *Xanthomonas campestris* pv. *vesicatoria*, revealing a coordinated hypersensitive response characterized by significant modulation of photosynthetic activity and abiotic stress-responsive genes. A study emphasized that the upregulation of PAL alongside other genes involved in lignin and carotenoid biosynthesis. Our transcriptome analysis showed that many genes involved in the phenylpropanoid biosynthesis pathway were significantly upregulated in ‘Parson Brown’. This leads to enhanced production of phenolic and flavonoid compounds, which fortify cell walls and provide antimicrobial activity. Also, stress-response pathways, including those regulating salicylic acid and jasmonic acid signaling, were more active in ‘Parson Brown’ than in ‘Hamlin’ and ‘Roble’. Killiny et al. [47] suggested that phenolic compounds, including ferulic acid and quinic acid, may contribute to Sugar Belle’s tolerance to HLB.

Flavonoids are vital secondary metabolites that contribute to plant defense through multiple complementary mechanisms. Flavonoids play a dual role by acting as antioxidants to neutralize ROS [45, 48]. Primarily, they act as antioxidants by scavenging ROS such as hydrogen peroxide (H₂O₂) and superoxide anion (O_2_^•−^), which are rapidly produced during pathogen invasion as part of the oxidative burst. While ROS serve as crucial signaling molecules that activate downstream defense pathways, their excessive accumulation can cause cellular oxidative damage, leading to membrane lipid peroxidation, protein oxidation, and DNA damage. Flavonoids maintain redox balance by neutralizing these harmful ROS, thereby protecting host cells from self-inflicted oxidative stress while preserving the signaling functions of ROS necessary for defense activation. Beyond their antioxidant role, flavonoids directly contribute to pathogen inhibition through antimicrobial activities [49, 50]. These include disrupting pathogen membrane integrity, chelating essential metals needed for pathogen survival, and modulating plant defense gene expression.

The infection with ‘*Ca*. L. asiaticus’ involves phloem blockage and nutrient depletion [51–53]. In the phloem, ‘*Ca*. L. asiaticus’ attaches to the plasma membrane at the sieve pores, triggering the expression of Callose Synthase and Phloem Protein 2 genes [54, 55]. This leads to the deposition of callose, which blocks the sieve pores and thickens the phloem cells, limiting nutrient transport and carbohydrate [53]. Although callose is widely recognized as a resistance-related barrier, its overaccumulation can become detrimental, as observed in susceptible cultivars, where it occurs along with starch buildup and tissue dysfunction. In our study, the susceptible cultivars ‘Hamlin’ and ‘Roble’ exhibited high levels of both callose deposition and starch accumulation. In contrast, the tolerant cultivar ‘Parson Brown’ showed significantly lower accumulation of these substances and exhibited upregulation of multiple glucan 1,3-beta-glucosidase genes.

Callose, a β−1,3-glucan polysaccharide, is typically synthesized at pathogen attack sites to limit pathogen spread [56], while β−1,3-glucanases hydrolyze these glucans, facilitating the breakdown of callose and related structures [57]. The significant upregulation of β−1,3-glucanases in ‘Parson Brown’ suggests an adaptive mechanism for managing phloem integrity by selectively degrading callose to prevent persistent occlusion and prolonged resource starvation. Callose degradation may increase sugar availability, which could serve as a carbon source for both pathogens and phloem sap-sucking insects such as the Asian citrus psyllid [18, 58]. We recorded an increase in disaccharide levels; however, these sugars are less accessible or poorly utilized by ‘*Ca*. L. asiaticus’, which depends primarily on host-derived monosaccharides such as glucose, fructose, and xylose for energy via glycolysis. These sugars are unlikely to be limiting factors for the growth and multiplication of ‘*Ca*. L. asiaticus’. While sugars do not directly contribute to plant defense against pathogens, their accumulation can activate pathogenesis-related proteins and enhance plant defense responses during infection [59].

HLB is a complex disease, and pathogen nutrition is supported by multiple metabolic pools beyond callose-derived sugars. For instance, ‘*Ca*. L. asiaticus’ infection markedly reduces concentrations of key tricarboxylic acid (TCA) cycle intermediates such as succinate, fumarate, citrate, and isocitrate in the phloem sap of infected *Madagascar periwinkle* and sweet orange ‘Valencia’ [60]. Killiny [60] proposed two non-mutually exclusive hypotheses to explain this reduction: (i) Phloem collapse or blockage impairs translocation, and (ii) *‘Ca.* L. asiaticus’ directly utilizes these intermediates to fulfill its nutritional requirements. Regarding the first hypothesis, phloem collapse and blockage are well-documented consequences of *‘Ca.* L. asiaticus’ infection, resulting in sieve pore plugging by phloem proteins and callose deposition [61–64]. This structural impairment limits the translocation of photosynthates from source leaves to sink tissues [65]. While callose occlusion is a common defense mechanism, it may not fully seal sieve plates or plasmodesmata [66]. The second hypothesis is supported by genomic analyses. The genome of ‘*Ca*. L. asiaticus’ is relatively small (~ 1.2 Mb) and lacks biosynthetic pathways for many essential amino and organic acids [3, 67, 68]. However, it encodes over 90 transporter genes, including more than 40 ATP-binding cassette (ABC) transporters, reflecting a heavy reliance on host-derived metabolites. Additionally, ‘*Ca*. L. asiaticus’ lacks genes for isocitrate lyase and malate synthase, making it incapable of synthesizing key TCA intermediates [67]. It is therefore believed that the bacterium scavenges host-derived succinate, fumarate, malate, and aspartate to support its TCA cycle and pyruvate production [35].

Interestingly, infected ‘Parson Brown’ trees exhibited a significant increase in the sugar alcohols chiro-inositol and scyllo-inositol, a trend not observed in ‘Hamlin’ or ‘Roble’. Certain Gram-negative α-proteobacteria, such as *Cronobacter*, are capable of utilizing sugar alcohols like sorbitol, mannitol, and inositol as carbon sources [69, 70], however, the ability of ‘*Ca*. L. asiaticus’ to utilize these sugar alcohols remains undocumented [18]. Thus, the combination of elevated disaccharides and reduced monosaccharide availability may limit carbon access and contribute to restricting bacterial growth in ‘Parson Brown’. This reduction is an adaptive response to restrict the availability of essential resources for bacterial proliferation.

‘Parson Brown’ showed significantly lower levels of H₂O₂ and O₂⁻, along with reduced DPPH scavenging activity, despite exhibiting the strongest tolerance phenotype. This suggests that ‘Parson Brown’ may possess a more efficient ROS homeostasis system, preventing prolonged oxidative stress and tissue damage. Rather than relying on a strong oxidative burst, its defense strategy may involve early containment of ROS through rapid activation of antioxidant genes, tight control of ROS-producing pathways, or ROS-independent defense pathways such as phenolic and flavonoid accumulation, which we observed at elevated levels. Controlled ROS regulation has been linked to durable resistance in several perennial crops, where limiting self-inflicted damage is critical under chronic infection conditions such as HLB. The observed differential expression patterns of glutathione S-transferase-like (GST) genes among the three citrus varieties suggest distinct antioxidant defense strategies in response to ‘*Ca.* L. asiaticus’ infection. The higher expression of specific GST homologs in ‘Parson Brown’ indicates a strong enzymatic detoxification capacity, potentially contributing to its enhanced tolerance by facilitating the conjugation and removal of ROS and toxic compounds. Similarly, the variation in thioredoxin-like protein gene expression points to unique redox regulation mechanisms.

We found that ‘Parson Brown’ emits specific VOCs that appear to deter psyllid landing, reducing opportunities for ‘*Ca*. L. asiaticus’ transmission. ‘Parson Brown’ exhibited significantly higher production of VOCs, such as terpenes and aldehydes, following infection. Killiny et al. [71] identified the main volatiles in ‘Sugar Belle’, a tolerant mandarin variety, as linalool, thymol, γ-terpinene, β-elemene, z-β-ocimene, and α-pinene. Furthermore, Killiny et al. [72] also found that ‘Sugar Belle’ mandarin on Carrizo rootstock had elevated levels of thymol and its precursors (γ-terpinene and p-cymene). In our previous study [10], we quantified oil content and limonoids, including limonin and nomilin, in ‘Parson Brown’ juice and found these compounds to be higher than in ‘Hamlin’ [10]. While the present study did not directly assess the relationship between VOC biosynthesis and oil content, this association suggests a possible link that worse further investigation. Although these compounds caused a bitter taste, a phenomenon observed in citrus fruit from HLB-affected trees [73], these compounds also contribute to resistance against pests and pathogens when released [74–76]. Additionally, Yuan et al. [77] reported that terpenoid volatiles, like α-terpinene, α-pinene, β-phellandrene, caryophyllene, and myrcene, influence the behavior of herbivorous insects on host plants.

To conclude, ‘Parson Brown’ exhibits a multifaceted tolerance to *Ca. L. asiaticus* is driven by extensive transcriptional reprogramming that drives metabolic adaptations and vector deterrence.

## Supplementary Information

Supplementary Material 1.

## Data Availability

The datasets presented in this study are available in online repositories. The repository name and accession details are as follows: BioProject-NCBI, ID: PRJNA1192728. https://dataview.ncbi.nlm.nih.gov/object/PRJNA1192728?reviewer=8ej298r4br1m6glar347pq3mnn.

## Abbreviations

HLB: Huanglongbing
‘*Ca*. L. asiaticus’: Candidatus Liberibacter asiaticus
Ct: Cycle Threshold
DEG(s): Differentially Expressed Gene(s)
FDR: False Discovery Rate
GO: Gene Ontology
KEGG: Kyoto Encyclopedia of Genes and Genomes
DPPH: 2,2-diphenyl-1-picrylhydrazyl
EDTA: Ethylenediaminetetraacetic Acid
H₂O₂: Hydrogen Peroxide
H2DCFDA: 2’,7’-dichlorodihydrofluorescein diacetate
IOD: Integrated Optical Density
NBT: Nitro Blue Tetrazolium
OD: Optical Density
O₂•−: Superoxide Anion
PBS: Phosphate Buffered Saline
qPCR: Quantitative Polymerase Chain Reaction
RT-qPCR: Reverse Transcription Quantitative PCR
ROS: Reactive Oxygen Species
POD: Peroxidase
GST: Glutathione S-transferase
SOD: Superoxide Dismutase
VOC(s): Volatile Organic Compound(s)
TMS: Trimethylsilyl derivatization
