# Supplementary material for: Multi-omics analyses reveal the defense mechanisms behind the tolerance of the ‘Parson Brown’ sweet orange to Huanglongbing
Source: BMC Plant Biol. 2025 Oct 3;25:1303. doi: 10.1186/s12870-025-07372-2 (PMC12495878; doi:10.1186/s12870-025-07372-2)
Supplement: Supplementary file 1 — Supplementary Material 1. [file 12870_2025_7372_MOESM1_ESM.docx]

**Multi-Omics Analyses Reveal the Defense Mechanisms behind the Tolerance of Sweet Orange Survivors to Citrus Greening Disease**

Lamiaa M. Mahmoud^1,3^, Shelley E Jones^1^, Pedro Gonzalez-Blanco^1^, Yu Fahong^2^, Manjul Dutt^3,4^, Nabil Killiny^1^*

^1^Department of Plant Pathology, Citrus Research and Education Center, University of Florida, Lake Alfred, USA

^2^Interdisciplinary Center for Biotechnology Research (UF | ICBR) at the University of Florida

^3^Department of Horticultural Sciences, Citrus Research and Education Center, University of Florida, Lake Alfred, FL, USA

^4^ Plant Breeding Graduate Program, University of Florida, Gainesville, Florida, USA

*Corresponding Author: Nabil Killiny,

Associate Professor

Department of Plant Pathology, Citrus Research and Education Center, IFAS, University of Florida, 700 Experiment Station Road, Lake Alfred, FL, 33850, USA

Email: [nabilkilliny@ufl.edu](mailto:nabilkilliny@ufl.edu)

Phone: 863-956-8833

Short title: HLB Tolerance in Sweet Orange Varieties

**Supplementary Table 1.** List of the primer sequences used SYBR Green based qPCR assay.

| Gene ID | Function | Sequence (5’ – 3’) |  |
| --- | --- | --- | --- |
| cs7g07640 | ChaC-like protein | GGA GAA GGC CAT GTT TGA TAT TG | CAA CCA ACT TCC TCT CCT TGA |
| cs3g07110 | Sieve element occlusion N-terminus | ACA CAG TTT ACC ATC CCA CTT T | GGG TCA AGA ACT ACC AGG ATT G |
| orange1.1t01815 | LRR_typ_2 | GAG CAA AGA TGA ATG GGA AAG TG | CTT CTG AGA GTC ATC CAA GCT ATC |
| cs3g26100 | Gibberellin regulated protein | TCT CGT TGA AGC TGA GCA TAC | GGT TTG GCC TCG ACG ATA AT |
| cs7g17630 | Glutathione Transferase | TGG AAC AAT GGC TGG AAG TAG | AAT TCT CAT CGG CAG GAA GTC |
| cs5g16700 | Sucrose synthase | TCT TGC CGC TCA CCT TTA C | AAA GGA AGA TTG TTT GGG CTA ATG |
| cs5g07550 | Beta-Amylase 3, Chloroplastic | TAG CAC GAA TGC TGG CTA AA | CCG AAC TAA TCC TTC TGG TGA G |
| cs7g03460 | RuBisCo LSMT C-terminal, substrate-binding domain | TCA GAG ATT GGA AGC TTG TGT AG | GAT AAA CCC GCC AAG GAG AA |
| cs5g23630 | FKBP-like | GAT GAC GCT TGC GAG AAA GA | GGG CCT TGA CCA ACT TTG ATA |
| cs3g12550 | DMP3 | TAC CAC TGG CCT ACA CAT CTT | CCT CCT TCT CAG TAC CCT TGT |
| cs1g19890 | Peroxidase | CGT TAA TGG TTG TGA TGC TTC TAT G | CCT GGA CAG GCT TTC TCT AAT G |
| cs9g01710 | Glycogenin subfamily member | AGT ACC CTC TTG TAG TTG CTA TTT | GGT AGA CCG GCT CGA TTT C |

**Supplementary Table 2.** Summary of sequencing, cleaning, and mapping of reads following sequencing three sweet oranges varities (‘Hamlin’, ‘Robles’ and ‘Parson brown’) under control and ‘*Ca*. L. asiaticus’ infection.

| **Sample name*** | **Raw-reads^a^** | **Clean_reads^b^** | **Uniq_mapped** | **%_Uniq_mapped** | **Mapped_transcripts** | **Total_splices** | **%_Unmapped** |
| --- | --- | --- | --- | --- | --- | --- | --- |
| **H-H-R1** | 27,415,584 | 27,316,404 | 25,006,603 | 91.54% | 20,877 | 24,015,610 | 3.14% |
| **H-H-R2** | 32,146,879 | 32,033,001 | 29,522,937 | 92.16% | 20,699 | 29,178,880 | 2.89% |
| **H-H-R3** | 30,165,598 | 30,098,813 | 27,999,412 | 93.02% | 21,028 | 26,472,928 | 1.85% |
| **H-I-R1** | 26,038,671 | 25,963,541 | 23,469,413 | 90.39% | 21,218 | 21,714,865 | 2.42% |
| **H-I-R2** | 29,989,309 | 29,869,549 | 27,278,776 | 91.33% | 21,646 | 24,894,609 | 3.41% |
| **H-I-R3** | 21,136,489 | 21,041,943 | 19,173,240 | 91.12% | 21,110 | 17,460,635 | 3.60% |
| **PB-H-R1** | 24,417,609 | 24,322,574 | 22,063,486 | 90.71% | 20,598 | 20,268,381 | 3.02% |
| **PB-H-R2** | 23,718,259 | 23,640,283 | 21,745,167 | 91.98% | 20,471 | 21,289,146 | 2.75% |
| **PB-H-R3** | 31,329,830 | 31,202,202 | 28,518,207 | 91.40% | 21,028 | 27,652,270 | 3.15% |
| **PB-I-R1** | 31,596,311 | 31,491,956 | 29,171,185 | 92.63% | 21,014 | 28,327,343 | 2.18% |
| **PB-I-R2** | 30,026,219 | 29,911,452 | 26,964,536 | 90.15% | 21,564 | 23,387,344 | 3.16% |
| **PB-I-R3** | 25,004,301 | 24,920,405 | 22,649,941 | 90.89% | 21,250 | 19,860,238 | 2.36% |
| **R-H-R1** | 24,096,330 | 23,999,815 | 21,601,730 | 90.01% | 20,554 | 20,666,191 | 2.88% |
| **R-H-R2** | 29,906,144 | 29,773,054 | 27,308,931 | 91.72% | 20,787 | 26,162,114 | 3.21% |
| **R-H-R3** | 16,952,059 | 16,911,690 | 15,545,295 | 91.92% | 20,447 | 14,673,891 | 2.14% |
| **R-I-R1** | 21,565,905 | 21,503,628 | 19,773,047 | 91.95% | 21,172 | 18,158,676 | 2.19% |
| **R-I-R2** | 23,819,625 | 23,724,420 | 21,611,410 | 91.09% | 21,286 | 19,757,287 | 3.00% |
| **R-I-R3** | 27,949,754 | 27,883,009 | 25,726,023 | 92.26% | 21,316 | 24,015,014 | 1.99% |

***** H: ‘Hamlin’, R: ‘Robles’, PB: ‘Parson brown’, H: control healthy conditions, and I: ‘*Ca*. L. asiaticus’ infection. Each variety was sequenced into three biological replicates.

**^a, b^** represents an average for two technical replicates for each sample.

**Supplementary Table 3.** Emitted volatile organic compounds (VOCs) from leaves of ‘Hamlin’, ‘Roble’ and ‘Parson brown’ under control and ‘*Ca*. L. asiaticus’ infection.

| **ID** | **RT*** | **Hamlin-Healthy** | **Hamlin- infected** | **Roble-**  **Healthy** | **Roble- infected** | **Parson Brown-**  **Healthy** | | **Parson Brown- infected** | **ANOVA** |
| --- | --- | --- | --- | --- | --- | --- | --- | --- | --- |
| **α-Pinene** | 8.1 | 6.68 ± 1.68 | 9.78 ± 4.74 | 6.96 ± 2.22 | 8.36 ± 4.73 | | 7.73 ± 1.94 | 10.66 ± 2.42 | ns |
| **Sabinene** | 8.78 | 137.57 ± 21.54 | 190.79 ± 89.67 | 137.31 ± 51.83 | 157.08 ± 88.9 | | 117.20 ± 67.95 | 205.64 ± 40.43 | ns |
| **β-Pinene** | 9.05 | 4.91 ± 1.37 | 8.28 ± 4.31 | 6.16 ± 3.93 | 7.75 ± 5.95 | | 6.89 ± 2.62 | 10.30 ± 3.53 | ns |
| **Myrcene** | 9.32 | 17.90 ± 3.33 | 18.35 ± 13.13 | 22.63 ± 2.71 | 15.36 ± 7.4 | | 17.23 ± 9.24 | 28.32 ± 6.82 | ns |
| **δ-Carene** | 9.77 | 23.32 ± 6.85 | 27.97 ± 14.49 | 41.82 ± 6.53 | 42.68 ± 16 | | 35.13 ± 3.6 | 45.83 ± 9.82 | **0.0140** |
| **α-Terpinene** | 10 | 0.00 ± 0 | 0.00 ± 0 | 0.00 ± 0 | 0.00 ± 0 | | 0.80 ± 1.1 | 1.22 ± 1.16 | **0.0183** |
| **p-Cymene** | 10.15 | 0.67 ± 0.12 | 0.67 ± 0.35 | 1.09 ± 0.19 | 1.01 ± 0.32 | | 0.88 ± 0.09 | 1.18 ± 0.24 | **0.0082** |
| **d-Limonene** | 10.25 | 17.54 ± 7.76 | 17.05 ± 4.17 | 22.86 ± 15.08 | 32.31 ± 29.11 | | 13.24 ± 12.51 | 34.02 ± 13.14 | ns |
| **β-Phellandrene** | 10.3 | 1.86 ± 1.24 | 2.38 ± 1.34 | 2.98 ± 0.71 | 3.50 ± 1.69 | | 3.28 ± 0.98 | 3.74 ± 1.11 | ns |
| **trans-β-Ocimene** | 10.63 | 10.54 ± 3.38 | 21.94 ± 12.7 | 17.84 ± 3.99 | 19.22 ± 9.61 | | 15.31 ± 4.52 | 21.17 ± 4.08 | ns |
| **γ-Terpinene** | 10.87 | 2.23 ± 0.4 | 2.76 ± 1.3 | 1.65 ± 1.09 | 2.20 ± 1.15 | | 2.32 ± 0.5 | 2.86 ± 0.51 | ns |
| **Sabinene hydrate** | 11 | 0.00 ± 0 | 0.00 ± 0 | 2.98 ± 0.88 | 2.37 ± 1.47 | | 1.83 ± 0.69 | 3.45 ± 0.58 | **<.0001** |
| **α-Terpinolene** | 11.32 | 0.79 ± 0.22 | 0.91 ± 0.43 | 1.30 ± 0.22 | 1.31 ± 0.52 | | 1.06 ± 0.11 | 1.39 ± 0.29 | **0.0415** |
| **Linalool** | 11.85 | 2.26 ± 1.01 | 8.08 ± 12.36 | 24.80 ± 5.87 | 23.83 ± 14.26 | | 20.18 ± 6.94 | 37.85 ± 8.99 | **<.0001** |
| **Nonanal** | 11.93 | 8.55 ± 0.66 | 7.80 ± 4.38 | 8.91 ± 2.79 | 6.74 ± 3.25 | | 10.23 ± 1.67 | 8.44 ± 1.91 | ns |
| **allo-Ocimene** | 12.45 | 0.00 ± 0 | 0.16 ± 0.16 | 0.00 ± 0 | 0.22 ± 0.22 | | 0.00 ± 0 | 0.00 ± 0 | **0.0099** |
| **Citronellal** | 13 | 6.04 ± 3.57 | 9.28 ± 9.45 | 29.23 ± 10.66 | 22.92 ± 9.22 | | 17.60 ± 7.02 | 34.59 ± 16.04 | **0.0008** |
| **Octanoic acid** | 13.25 | 2.82 ± 3.17 | 1.18 ± 0.65 | 1.68 ± 0.4 | 1.35 ± 0.59 | | 1.67 ± 0.22 | 1.60 ± 0.37 | ns |
| **α-Terpineol** | 13.95 | 0.00 ± 0 | 0.00 ± 0 | 0.71 ± 0.5 | 0.47 ± 0.4 | | 0.25 ± 0.29 | 0.55 ± 0.11 | **0.0024** |
| **Decanal** | 14.1 | 0.15 ± 0.21 | 0.24 ± 0.23 | 0.40 ± 0.24 | 0.89 ± 0.39 | | 0.31 ± 0.47 | 1.03 ± 0.53 | **0.0029** |
| **β-Citronellol** | 14.5 | 1.87 ± 1.35 | 3.24 ± 2.81 | 2.59 ± 0.78 | 2.31 ± 0.66 | | 2.52 ± 1.16 | 3.25 ± 0.66 | ns |
| **Neral** | 14.75 | 6.22 ± 4.17 | 10.87 ± 8.53 | 36.37 ± 29.1 | 25.74 ± 10.64 | | 18.51 ± 2.8 | 46.56 ± 18.37 | **0.0025** |
| **Geraniol** | 15 | 0.75 ± 0.45 | 1.07 ± 1.83 | 2.78 ± 1.37 | 1.19 ± 0.75 | | 3.77 ± 2.28 | 2.75 ± 0.62 | **0.0109** |
| **Nonanoic acid** | 15.25 | 2.83 ± 2.99 | 7.77 ± 15.31 | 6.37 ± 2.71 | 2.06 ± 4.34 | | 3.00 ± 3.03 | 7.28 ± 0.98 | ns |
| **Geranial** | 15.35 | 10.73 ± 10.11 | 13.69 ± 8.17 | 42.93 ± 23.65 | 29.98 ± 16.08 | | 30.99 ± 3.13 | 43.28 ± 14.11 | **0.0032** |
| **Geranic acid methyl ester** | 16.4 | 0.00 ± 0 | 3.62 ± 4.96 | 0.71 ± 1.6 | 1.48 ± 2.17 | | 0.69 ± 1.54 | 2.01 ± 1.84 | ns |
| **δ-Elemene** | 16.764 | 0.19 ± 0.07 | 0.18 ± 0.08 | 0.49 ± 0.25 | 0.43 ± 0.3 | | 0.41 ± 0.19 | 0.72 ± 0.21 | **0.0027** |
| **Citronellyl acetate** | 16.9 | 0.73 ± 0.59 | 1.72 ± 2.16 | 3.23 ± 1.44 | 1.81 ± 0.99 | | 1.55 ± 1.12 | 2.33 ± 1.12 | 0.1186 |
| **Neryl acetate** | 17.1 | 0.24 ± 0.15 | 0.71 ± 0.94 | 0.22 ± 0.38 | 0.41 ± 0.52 | | 0.35 ± 0.36 | 0.21 ± 0.31 | ns |
| **Geranyl acetate** | 17.44 | 0.33 ± 0.29 | 0.65 ± 0.91 | 4.89 ± 3.9 | 2.24 ± 1.03 | | 1.72 ± 1.25 | 3.16 ± 1.46 | **0.0076** |
| **γ-Cadinene** | 17.51 | 0.14 ± 0.08 | 0.16 ± 0.04 | 0.15 ± 0.1 | 0.16 ± 0.06 | | 0.09 ± 0.03 | 0.17 ± 0.02 | ns |
| **α-Elemene** | 0 | 1.11 ± 0.15 | 1.19 ± 0.5 | 1.16 ± 0.91 | 1.07 ± 0.66 | | 0.97 ± 0.33 | 1.54 ± 0.43 | ns |
| **trans-β-Elemene** | 17.85 | 19.25 ± 2.52 | 20.45 ± 7.29 | 20.40 ± 16.01 | 18.77 ± 12.08 | | 16.79 ± 5.81 | 26.91 ± 7.09 | ns |
| **α-Bergamotene** | 18.31 | 0.24 ± 0.17 | 0.27 ± 0.05 | 0.20 ± 0.23 | 0.16 ± 0.22 | | 0.19 ± 0.17 | 0.35 ± 0.13 | ns |
| **trans-β-Caryophyllene** | 18.45 | 9.34 ± 1.11 | 10.86 ± 2.31 | 5.83 ± 2.22 | 6.57 ± 2.7 | | 5.22 ± 0.84 | 7.08 ± 1.03 | **0.0004** |
| **Aromadendrene** | 18.6 | 0.11 ± 0.15 | 0.00 ± 0 | 0.03 ± 0.06 | 0.00 ± 0 | | 0.10 ± 0.12 | 0.00 ± 0 | ns |
| **trans-β-Farnesene** | 18.85 | 3.95 ± 2.78 | 2.72 ± 1.85 | 2.18 ± 1.81 | 1.55 ± 1.32 | | 1.98 ± 1.44 | 2.75 ± 1.01 | ns |
| **β-Bergamotene** | 18.9 | 0.18 ± 0.25 | 0.44 ± 0.26 | 0.32 ± 0.41 | 0.45 ± 0.4 | | 0.45 ± 0.34 | 0.67 ± 0.22 | ns |
| **α-Humulene** | 19 | 2.51 ± 0.26 | 2.68 ± 0.58 | 2.33 ± 1.33 | 2.26 ± 1.15 | | 2.08 ± 0.49 | 2.95 ± 0.49 | ns |
| **Valencene** | 19.7 | 0.12 ± 0.11 | 0.15 ± 0.14 | 0.96 ± 0.7 | 0.66 ± 0.75 | | 0.21 ± 0.29 | 0.71 ± 0.47 | ns |
| **α-Farnesene** | 19.82 | 0.93 ± 0.27 | 0.70 ± 0.75 | 0.88 ± 0.82 | 0.68 ± 0.38 | | 0.25 ± 0.36 | 0.54 ± 0.57 | ns |
| **Elemol** | 20.6 | 0.41 ± 0.24 | 0.56 ± 0.11 | 0.40 ± 0.42 | 0.44 ± 0.34 | | 0.32 ± 0.3 | 0.66 ± 0.14 | ns |
| **Dodecanoic acid** | 20.7 | 2.46 ± 0.52 | 1.44 ± 1.01 | 2.65 ± 0.91 | 1.15 ± 0.55 | | 2.32 ± 0.4 | 1.67 ± 0.8 | **0.0163** |
| **β-Sinensal** | 23 | 268.16 ± 50.12 | 263.77 ± 51.51 | 306.07 ± 186.59 | 279.94 ± 227.6 | | 288.21 ± 141.01 | 386.04 ± 136.16 | ns |
| **α-Sinensal** | 23.85 | 42.88 ± 6.08 | 28.14 ± 23.68 | 29.30 ± 40.28 | 40.18 ± 26.34 | | 41.32 ± 25.1 | 26.00 ± 11.98 | ns |
| **Tetradecanoic acid** | 24 | 82.77 ± 10.73 | 37.97 ± 22.56 | 69.40 ± 18.05 | 26.76 ± 11.78 | | 74.21 ± 8.37 | 40.33 ± 19.24 | **<.0001** |
| **Hexadecanoic acid** | 27 | 193.34 ± 18.97 | 124.65 ± 51.26 | 160.45 ± 45.49 | 72.44 ± 26.01 | | 178.75 ± 35.4 | 115.62 ± 50.43 | **0.0007** |
| **Phytol** | 29 | 97.86 ± 26.85 | 157.76 ± 45.39 | 84.10 ± 34.29 | 136.15 ± 45.75 | | 91.43 ± 53.59 | 123.24 ± 28.53 | ns |
| **Total** |  | 993 ± 198. | 1059 ± 474 | 1118 ± 525 | 1006 ± 590 | | 1041 ± 413 | 1302 ± 410 | ns |

VOCs were detected using headspace solid-phase microextraction and gas chromatography-mass spectrometry (GC-MS). *P*-values < 0.05 indicate significant differences (*n* = 5).

*RT signifies Retention time.

VOCs were initially identified using Wiley 9th ed., NIST 2011, and Wiley Flavor and Fragrance mass spectral libraries.

**Supplementary Table 4.** Content of Metabolite in leaves of ‘Hamlin’, ‘Roble’ and ‘Parson brown’ under control and ‘*Ca*. L. asiaticus’ infection.

|  | **Hamlin** | | | **Parson Brown** | | | **Roble** | | |
| --- | --- | --- | --- | --- | --- | --- | --- | --- | --- |
| **TMS Metabolite** | **Healthy** | **Infected** | ***p* value** | **Heathy** | **Infected** | ***p* value** | **Healthy** | **Infected** | ***p* value** |
| Glycolic acid | 0.06 ± 0.14 | 0.28 ± 0.31 | ns | 0.04 ± 0.04 | 0.10 ± 0.14 | ns | 0.08 ± 0.09 | 0.54 ± 0.47 | ns |
| L-Alanine | 1.79 ± 2.48 | 0.55 ± 0.44 | ns | 1.27 ± 1.16 | 2.35 ± 2.58 | ns | 0.42 ± 0.51 | 0.84 ± 0.14 | ns |
| L-valine | 0.05 ± 0.08 | 0.17 ± 0.25 | ns | 0.66 ± 1.05 | 0.03 ± 0.02 | ns | 0.17 ± 0.24 | 0.14 ± 0.24 | ns |
| Benzoic acid | 1.20 ± 1.97 | 0.67 ± 0.65 | ns | 11.02 ± 12.84 | 1.27 ± 2.00 | ns | 2.69 ± 2.78 | 1.60 ± 3.28 | ns |
| Phosphoric acid | 0.01 ± 0.02 | 0.12 ± 0.13 | ns | 0.11 ± 0.10 | 0.02 ± 0.02 | ns | 0.08 ± 0.13 | 0.06 ± 0.05 | ns |
| Glycerol | 0.59 ± 0.54 | 2.05 ± 2.19 | ns | 5.79 ± 3.73 | 0.45 ± 0.43 | 0.013 | 0.93 ± 1.35 | 0.27 ± 0.38 | ns |
| L-Proline | 7.60 ± 12.13 | 65.66 ± 88.40 | ns | 5.76 ± 3.98 | 1.27 ± 2.78 | ns | 2.00 ± 2.34 | 2.84 ± 4.20 | ns |
| Glycine | 0.00 ± 0.00 | 0.03 ± 0.02 | 0.012 | 0.01 ± 0.01 | 0.00 ± 0.00 | ns | 0.03 ± 0.06 | 0.00 ± 0.00 | ns |
| Succinic acid | 0.17 ± 0.21 | 0.57 ± 0.81 | ns | 0.26 ± 0.15 | 0.13 ± 0.09 | ns | 0.06 ± 0.09 | 0.34 ± 0.60 | ns |
| Fumaric acid | 0.00 ± 0.00 | 0.02 ± 0.02 | ns | 0.01 ± 0.01 | 0.00 ± 0.00 | ns | 0.02 ± 0.04 | 0.01 ± 0.01 | ns |
| L-Serine | 0.95 ± 1.24 | 4.93 ± 3.56 | 0.046 | 2.46 ± 2.11 | 0.86 ± 0.42 | ns | 0.47 ± 0.49 | 0.84 ± 0.73 | ns |
| L-Threonine | 0.12 ± 0.17 | 0.65 ± 0.64 | ns | 0.34 ± 0.45 | 0.07 ± 0.10 | ns | 0.03 ± 0.05 | 0.07 ± 0.17 | ns |
| 3-hydroxyvaleric acid | 0.32 ± 0.34 | 0.87 ± 0.87 | ns | 0.51 ± 0.32 | 0.16 ± 0.07 | 0.046 | 0.11 ± 0.11 | 0.19 ± 0.26 | ns |
| 2-Ketogluconic acid | 0.03 ± 0.02 | 0.14 ± 0.09 | 0.032 | 0.09 ± 0.08 | 0.04 ± 0.01 | ns | 0.23 ± 0.47 | 0.04 ± 0.02 | ns |
| Malic acid | 0.71 ± 0.69 | 3.66 ± 2.82 | ns | 11.58 ± 13.76 | 1.40 ± 0.41 | ns | 1.23 ± 1.87 | 3.44 ± 1.71 | ns |
| GABA | 6.79 ± 2.99 | 14.83 ± 10.57 | ns | 15.88 ± 6.71 | 6.33 ± 2.26 | 0.017 | 2.41 ± 1.73 | 5.15 ± 4.35 | ns |
| Threonic acid | 0.12 ± 0.07 | 2.12 ± 2.46 | ns | 0.29 ± 0.41 | 0.23 ± 0.08 | ns | 0.27 ± 0.42 | 0.67 ± 0.37 | ns |
| 2-Ketoglutaric acid | 0.86 ± 0.31 | 4.50 ± 2.90 | 0.024 | 1.55 ± 1.43 | 1.23 ± 0.51 | ns | 0.27 ± 0.20 | 0.82 ± 0.52 | ns |
| L-Phenylalanine | 0.09 ± 0.05 | 0.29 ± 0.22 | ns | 0.08 ± 0.02 | 0.09 ± 0.02 | ns | 0.02 ± 0.02 | 0.06 ± 0.06 | ns |
| Synephrine | 0.32 ± 0.13 | 2.39 ± 1.49 | 0.015 | 0.66 ± 0.56 | 0.41 ± 0.28 | ns | 0.23 ± 0.16 | 0.41 ± 0.24 | ns |
| Xylitol | 42.14 ± 16.49 | 75.93 ± 31.79 | ns | 50.78 ± 27.31 | 65.69 ± 8.78 | ns | 10.57 ± 5.11 | 24.59 ± 28.13 | ns |
| Citric acid | 28.45 ± 24.49 | 18.67 ± 15.35 | ns | 57.85 ± 26.96 | 2.18 ± 0.76 | 0.002 | 8.73 ± 6.89 | 3.34 ± 3.91 | ns |
| Iso-Citric acid | 0.64 ± 0.90 | 0.70 ± 0.79 | ns | 0.18 ± 0.14 | 0.33 ± 0.50 | ns | 0.02 ± 0.02 | 0.35 ± 0.35 | ns |
| Myristic acid | 0.46 ± 0.69 | 0.52 ± 0.78 | ns | 0.99 ± 0.46 | 0.10 ± 0.15 | 0.003 | 0.00 ± 0.00 | 0.00 ± 0.00 | ND |
| Quinic acid | 0.00 ± 0.00 | 1.11 ± 1.28 | ns | 0.02 ± 0.03 | 0.04 ± 0.04 | ns | 0.43 ± 0.91 | 17.45 ± 16.34 | 0.049 |
| Fructose | 12.29 ± 14.14 | 43.07 ± 28.34 | ns | 24.49 ± 22.92 | 19.89 ± 17.51 | ns | 6.62 ± 10.03 | 28.52 ± 16.25 | 0.033 |
| Mannose | 4.01 ± 5.11 | 3.14 ± 2.23 | ns | 8.12 ± 2.36 | 1.50 ± 0.99 | 0.000 | 0.75 ± 0.19 | 0.61 ± 0.90 | ns |
| Glucose | 22.03 ± 20.93 | 97.57 ± 98.51 | ns | 36.78 ± 35.16 | 39.11 ± 34.05 | ns | 12.47 ± 18.06 | 160.08 ± 100.41 | 0.012 |
| chiro-Inositol | 48.30 ± 41.31 | 42.69 ± 10.38 | ns | 84.35 ± 26.12 | 135.98 ± 16.59 | 0.006 | 23.88 ± 19.14 | 109.10 ± 43.48 | 0.004 |
| Glucaric acid | 0.24 ± 0.29 | 0.48 ± 0.48 | ns | 0.13 ± 0.05 | 0.05 ± 0.11 | ns | 0.08 ± 0.17 | 0.39 ± 0.24 | 0.044 |
| scyllo-Inositol | 15.69 ± 16.57 | 6.78 ± 3.43 | ns | 22.01 ± 6.36 | 26.93 ± 6.40 | ns | 2.13 ± 1.34 | 16.47 ± 11.95 | 0.029 |
| Palmitic acid | 7.47 ± 8.04 | 1.89 ± 1.94 | ns | 5.03 ± 5.17 | 1.53 ± 2.05 | ns | 0.51 ± 0.33 | 3.83 ± 3.92 | ns |
| myo-Inositol | 27.10 ± 25.69 | 21.76 ± 8.71 | ns | 13.21 ± 5.74 | 10.70 ± 4.92 | ns | 2.04 ± 1.43 | 15.70 ± 5.28 | 0.001 |
| Ferulic acid | 0.03 ± 0.07 | 0.04 ± 0.07 | ns | 0.02 ± 0.04 | 0.00 ± 0.00 | ns | 0.01 ± 0.02 | 0.06 ± 0.06 | 0.070 |
| Glucitol | 0.71 ± 0.74 | 1.81 ± 1.62 | ns | 0.20 ± 0.11 | 0.38 ± 0.45 | ns | 0.12 ± 0.21 | 2.12 ± 1.22 | 0.007 |
| Phytol | 16.30 ± 13.73 | 24.07 ± 18.63 | ns | 8.53 ± 2.30 | 6.35 ± 6.40 | ns | 1.21 ± 0.96 | 21.14 ± 12.73 | 0.008 |
| α-D-galactoside | 38.94 ± 31.82 | 69.81 ± 38.69 | ns | 61.10 ± 12.84 | 30.32 ± 12.20 | 0.005 | 5.76 ± 6.08 | 28.41 ± 21.02 | 0.049 |
| α-Linolenic acid | 67.72 ± 53.02 | 6.40 ± 5.43 | 0.033 | 48.93 ± 29.95 | 6.92 ± 8.21 | 0.016 | 15.49 ± 17.86 | 1.97 ± 2.13 | ns |
| Stearic acid | 2.91 ± 1.04 | 1.01 ± 1.26 | 0.032 | 4.52 ± 4.60 | 3.13 ± 3.45 | ns | 1.58 ± 2.10 | 2.63 ± 3.39 | ns |
| α-D-Galactoside, methyl ester | 2.87 ± 2.05 | 1.65 ± 0.69 | ns | 2.19 ± 1.29 | 0.85 ± 0.71 | ns | 1.37 ± 1.78 | 1.23 ± 0.94 | ns |
| Galacturonic acid | 0.00 ± 0.00 | 1.96 ± 1.26 | 0.008 | 0.92 ± 1.38 | 1.19 ± 1.18 | ns | 0.04 ± 0.09 | 1.76 ± 1.02 | 0.006 |
| Glucuronic acid | 1.55 ± 2.16 | 2.77 ± 2.04 | ns | 2.89 ± 3.34 | 1.42 ± 2.10 | ns | 1.34 ± 1.87 | 2.05 ± 1.10 | ns |
| Unk *m/z* 204/219 | 9.10 ± 9.52 | 7.41 ± 5.18 | ns | 5.14 ± 4.20 | 9.51 ± 9.99 | ns | 4.54 ± 6.49 | 3.63 ± 3.16 | ns |
| Sucrose | 526.71 ± 239.89 | 798.63 ± 342.08 | ns | 232.37 ± 44.59 | 398.45 ± 194.96 | ns | 183.94 ± 47.53 | 918.47 ± 88.22 | 0.000 |
| Maltose | 1.41 ± 1.88 | 1.95 ± 1.43 | ns | 1.51 ± 0.63 | 1.62 ± 1.17 | ns | 0.40 ± 0.38 | 1.36 ± 1.40 | ns |
| Melibiose | 4.38 ± 7.01 | 1.64 ± 2.21 | ns | 2.66 ± 2.82 | 2.71 ± 4.59 | ns | 0.24 ± 0.53 | 1.20 ± 2.57 | ns |
| β-Sitosterol | 1.29 ± 2.88 | 0.71 ± 0.98 | ns | 3.91 ± 3.97 | 2.73 ± 2.85 | ns | 0.77 ± 1.05 | 0.00 ± 0.00 | ns |
| **Total** | **904 ± 564** | **1,338 ± 744.** | **ns** | **737 ± 319** | **786 ± 353** | **ns** | **296 ± 163** | **1,384 ± 387** |  |
|  |  |  |  |  |  |  |  |  |  |

**
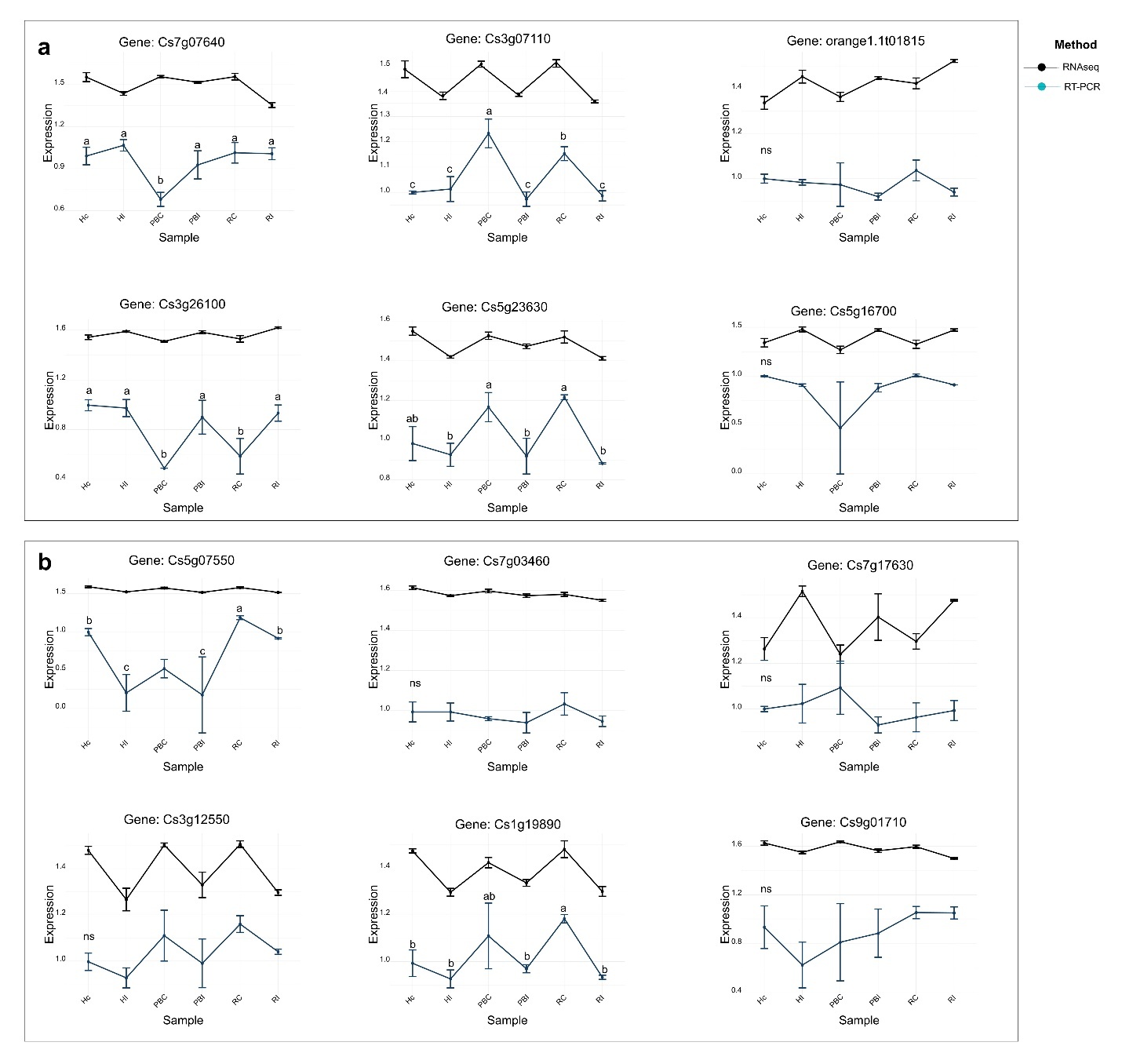
**

**Supplementary Fig. 1.** Verification of expression levels of selected upregulated **a** or downregulated **b** DEGs in ‘Hamlin’, ‘Roble’ and ‘Parson brown’ under control and ‘*Ca*. L. asiaticus’ infection as determined by RT-qPCR (2^-ΔΔCt^).Two-way ANOVA was performed on data obtained from RT-qPCR to assess the effects of variety, treatment (healthy vs. infected), and their interaction. Different letters indicate statistically significant differences based on Tukey’s post hoc test (p < 0.05).


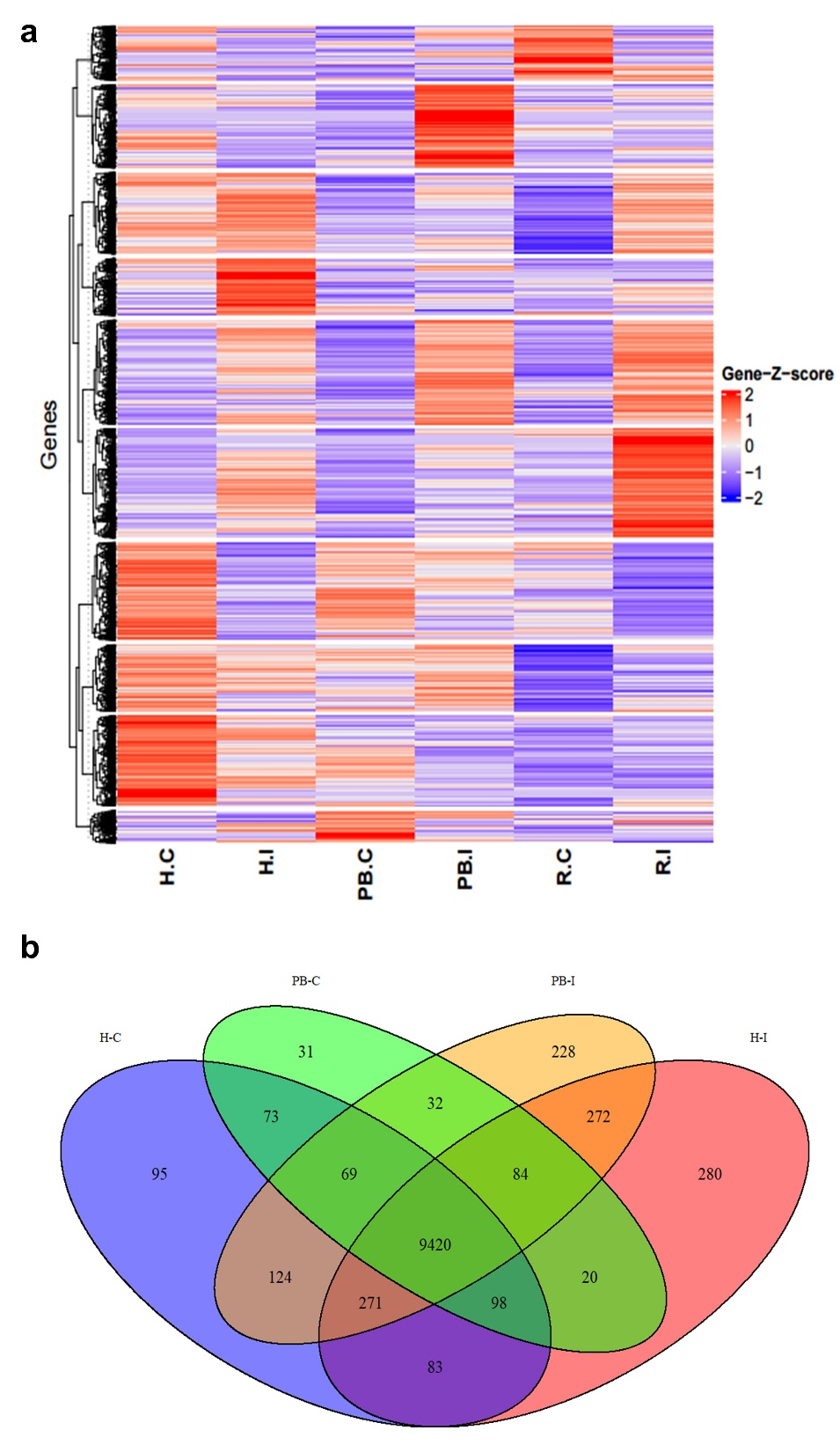


**Supplementary Fig. 2. a** Heat map representing gene expression profiles across all varieties under both control and infection conditions. **b** Venn diagram highlighting the shared and unique differentially expressed genes between ‘Hamlin’ and ‘Parson Brown’ under control and infection conditions.


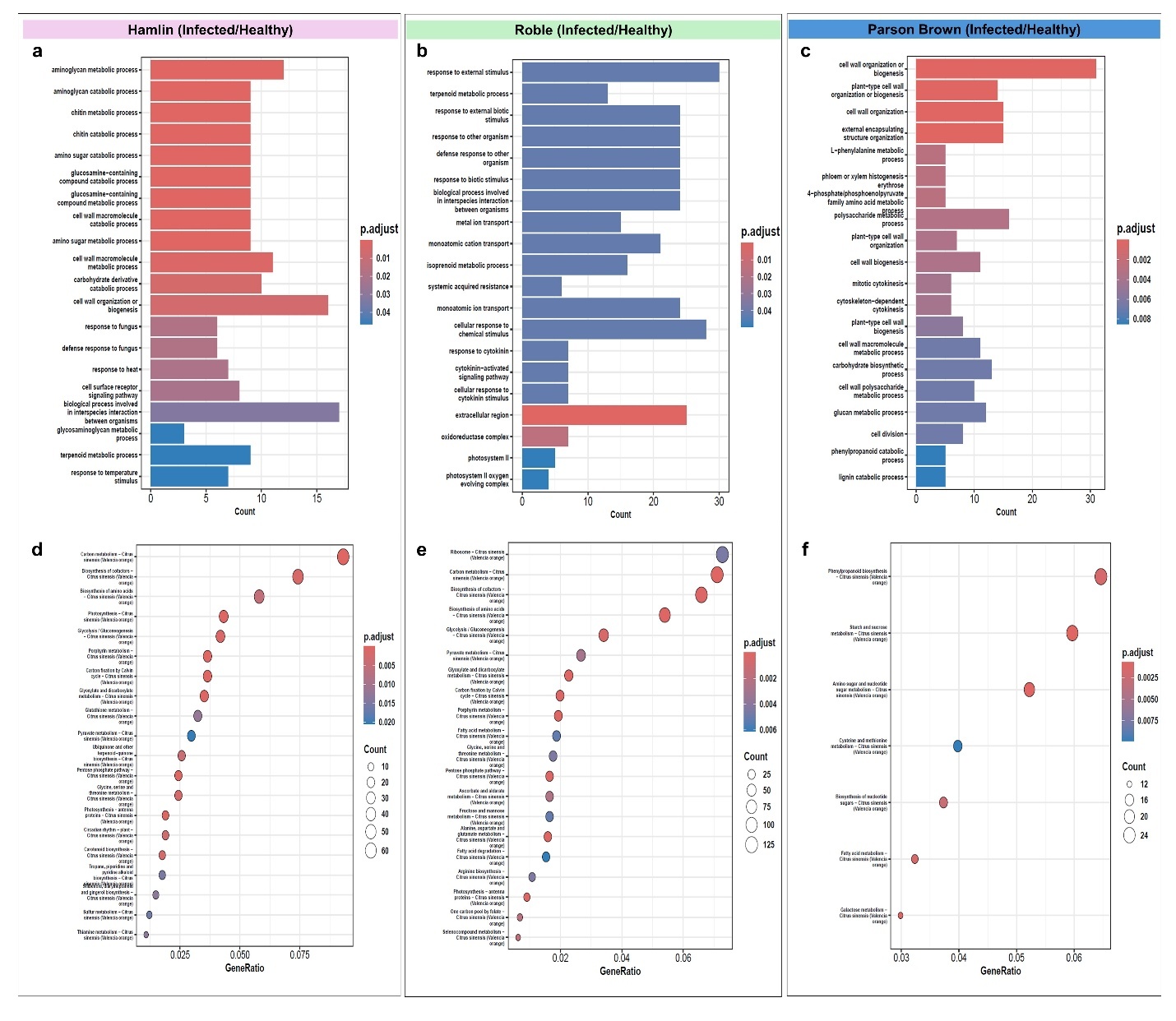


**Supplementary Fig. 3**. Differentially expressed genes (DEGs) and their functional enrichment across three sweet orange varieties (‘Hamlin’, ‘Parson Brown’, and ‘Roble’) under controlled conditions compared to the infected trees. Panels a, b, and c represent KEGG pathway analysis of DEGs specific to each comparison. Panels d, e, and f.


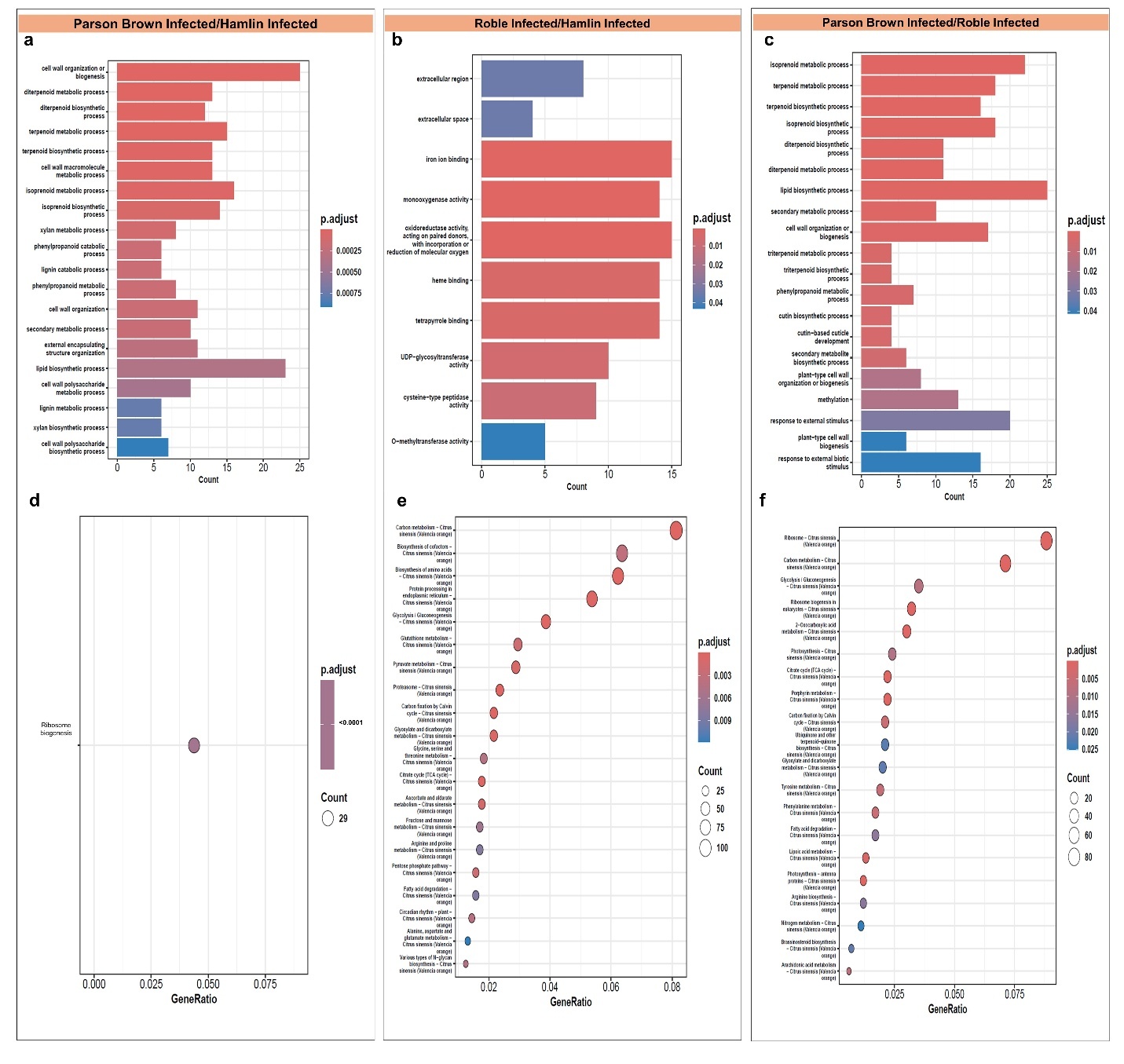


**Supplementary Fig. 4**. Differentially expressed genes (DEGs) and their functional enrichment across three sweet orange varieties (‘Hamlin’, ‘Parson Brown’, and ‘Roble’) following ‘*Ca*. L. asiaticus’ -infection compared to each other. Panels a, b, and c depict KEGG pathway analysis of DEGs specific to each comparison. Panels d, e, and f.


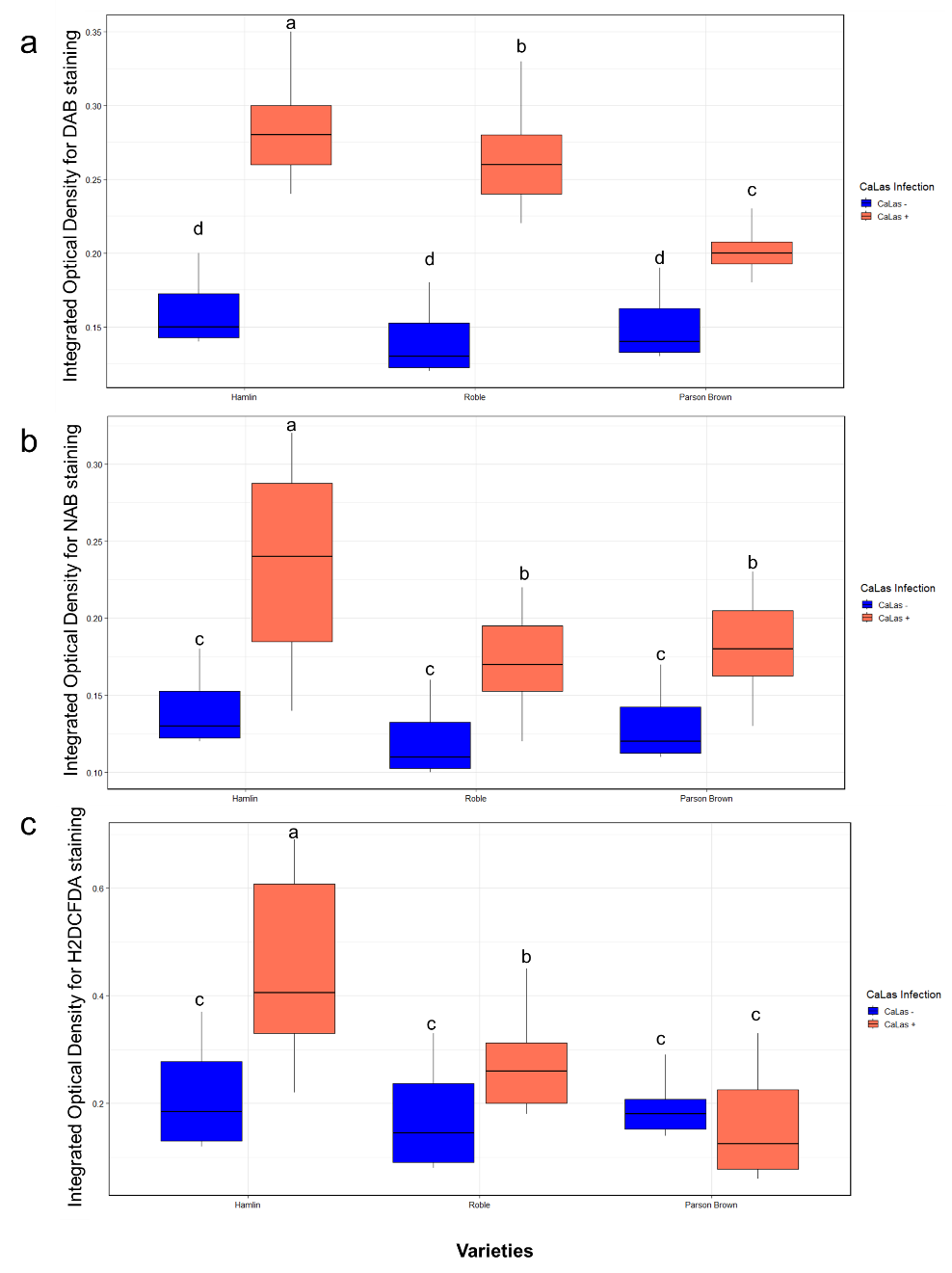


**Supplementary Figure 5.** *In situ* histochemical detection of reactive oxygen species (ROS) in citrus leaves. **a** integrated optical density (IOD) of DAB staining for hydrogen peroxide (H₂O₂) localization. **b** IOD of NBT staining for superoxide anion (O₂⁻). **c** IOD of H₂DCFDA staining fluorescence in leaves of ‘Hamlin’, ‘Parson Brown’, and ‘Roble’. Two-way ANOVA was performed to assess the effects of variety, treatment (healthy vs. infected), and their interaction. Different letters indicate statistically significant differences based on Tukey’s post hoc test (p < 0.05).
